# An Integrated Nanosensor/Smartphone Platform for Point-of-Care Biomonitoring of Human Exposure to Pesticides

**DOI:** 10.1021/acs.analchem.4c06421

**Published:** 2025-04-25

**Authors:** Hussian Maanaki, Letice Bussiere, Aleksandr Smirnov, Xiuxia Du, Yu Sun, Thomas A. Arcury, Phillip Summers, Landon Butler, Carey Pope, Anna Jensen, Gregory D. Kearney, Joshua T. Butcher, Jun Wang

**Affiliations:** 1 Department of Bioinformatics and Genomics, University of North Carolina at Charlotte, Charlotte, North Carolina 28223, United States; 2 Nanodiagnostic Technology, LLC, Kannapolis, North Carolina 28081, United States; 3 Wake Forest University School of Medicine, Winston-Salem, North Carolina 27157, United States; 4 Department of Physiological Sciences, Oklahoma State University, Stillwater, Oklahoma 74078, United States; 5 North Carolina Farmworkers Project, Benson, North Carolina 27504, United States; 6 Department of Public Health, East Carolina University, Greenville, North Carolina 27834, United States; 7 Center for Environmental monitoring and Informatics Technologies for Public Health, University of North Carolina at Charlotte, Charlotte, North Carolina 28223, United States

## Abstract

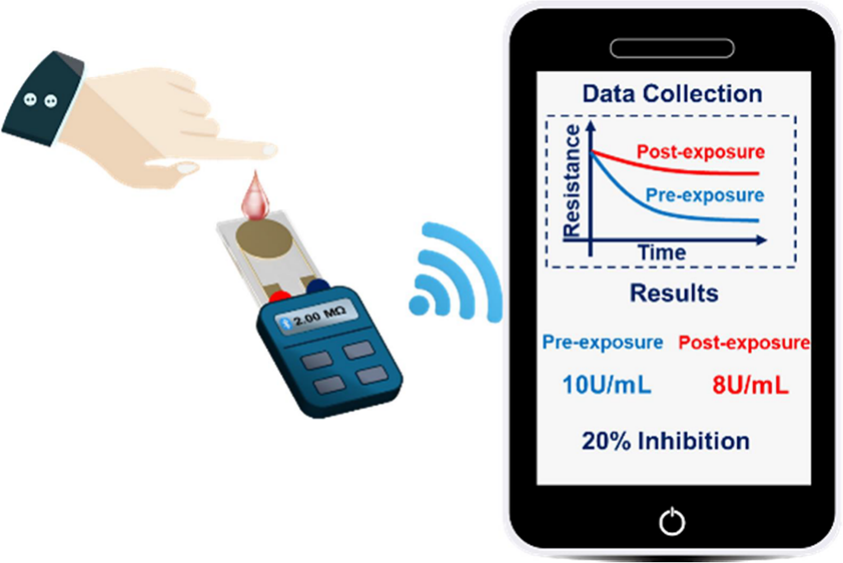

Organophosphorus (OP) compounds are neurotoxins that are among the most widely used pesticides in agriculture in the United States. In this application, a new integrated point-of-care smartphone/resistive nanosensor device is developed for onsite rapid and sensitive detection of exposure to OP pesticides from a drop of finger-stick blood among a sample of farmworkers. The nanosensor leverages the transport properties of a multiwalled carbon nanotube/polyaniline nanofiber (MWCNT/PAnNF) nanocomposite film on a gold interdigitated electrode and acetylcholinesterase/butyrylcholinesterase (AChE/BChE) hydrolysis of their respective substrates generating protons doping PAnNFs, thereby increasing the conductance of the film. As such, a conductance change can be used to quantify cholinesterase activity, enabling assessment of acute/chronic OP poisoning. Additionally, a mobile app was developed for the nanosensor to process, display, track, and share results. Under optimal conditions, the nanosensor demonstrated exceptional sensitivity with the detection limits of 0.11 U/mL for AChE and 0.093 U/mL for BChE, physiologically relevant dynamic ranges of 2.0–18.0 U/mL for AChE and 0.5–5.0 U/mL for BChE in whole blood, and high reproducibility with the relative standard variation of <4%. The nanosensor was further validated with widely used radiometric and Ellman’s methods, utilizing both *in vitro* pesticide-spiked blood samples and blood samples from 22 farmworkers. The results between this nanosensor and those two methods demonstrated a strong agreement. This platform provides a new avenue for the simple, rapid, and sensitive biomonitoring of OP pesticide exposure.

## Introduction

Exposure to organophosphate (OP) pesticides is a great public health concern across the world because of their neurotoxicity and widespread use in agriculture and landscaping.^1^ Mechanistically, exposure to OP pesticides on human and environmental health has been extensively studied.^2−4^ OP pesticides are known neurotoxicants due to their capabilities to irreversibly inhibit cholinergic esterases predominantly acetylcholinesterase (AChE) and form organo-phosphorylated AChE (OP-AChE), leading to a cholinergic crisis. Acute exposure to OP pesticides (>50% enzyme inhibition) can cause overaccumulation of acetylcholine (ACh), a neurotransmitter within the synapse and neuromuscular junction, leading to blurred vision, muscle spasms, paralysis, respiratory and cardiovascular symptoms, and even death.^5−9^ Chronic low-level exposure (<20% enzyme inhibition) does not demonstrate acute cholinergic symptoms, but increasing evidence suggests that it is highly associated with cognitive/behavioral deficits and neurological disorders in children.^4,10,11^ Furthermore, their association with diseases such as cancers,^12−14^ Parkinson’s disease,^15−17^ and chronic kidney disease has been documented.^18−21^

The primary biomarkers for assessment of OP pesticide exposure are through measurements of red blood cell AChE and plasma butyrylcholinesterase (BChE).^22^ Due to the short half-life of BChE, e.g., ∼12 days, BChE is used as a biomarker to assess recent exposure to OP pesticides. On the other hand, AChE has a longer half-life in the body, e.g., 33 days, making it a more useful biomarker to assess long-term exposure to OP pesticides. Ellman’s assay is the most widely used spectrophotometric method for determination of OP pesticide exposure, by measuring changes in blood cholinesterase (ChE) activity. Ellman’s method requires centralized laboratories with expensive instrumentation and trained personnel and is labor-intensive with a long turnaround time, leading to high costs.^23,24^ It is susceptible to wide statistical variation due to the presence of sulfhydryl groups (R-SH) and light absorption from hemoglobin within a sample matrix, which can interfere with the spectrophotometric signal.^25,26^ To add to its complexity, Ellman’s method necessitates multiple doctor visits for obtaining blood samples pre-exposure (i.e., baseline) and postexposure, resulting in limited temporal measurements and increased expense.

Other lab-centric techniques using chromatography coupled with mass spectrometry have also been developed for highly selective detection of OP compounds and their derivatives in urine, whole blood, and serum.^27,28^ Though these techniques are highly reliable and sensitive, they have numerous disadvantages, including high-cost instrumentation and complex sample preparation, and require trained personnel. The lack of informatics capabilities for lab-centralized diagnostics restricts the tracking and understanding of the health effects of chronic low-level OP pesticide exposure on population-level health, as the world steers toward mobile health^29^ and public biomonitoring.^30^

Given the limitations and lack of scalability for current methods, there is a need for portable, rapid, and sensitive point-of-care (POC) biomonitoring of OP pesticide exposure to improve population health and mitigate occupational/environmental health hazards among farmworkers and the general population. Biosensors have gained attention in recent decades due to their ability to detect various biomarkers with high sensitivity, short analytical time, low-cost, and simplicity of use. Integrating biosensors with mobile technologies and cloud-based infrastructure has made them an essential tool for the future of health^31^ and environmental monitoring.^32^ Their use in detecting pesticides in food and environmental water has been reported,^32−35^ and their application for biomonitoring of pesticide exposure has shown growing interest in recent years.^36,37^

Biosensors for assessing OP pesticide exposure consist of three major categories: (1) measuring blood ChE activity. These sensors have three primary modes of detection: (i) Hydrolysis of substrate (e.g., ACh/butyrylcholine (BCh)) to produce protons, which can be resistively measured (as demonstrated herein). (ii) Hydrolysis of substrate (e.g., acetylthiocholine (ATCh)) to produce thiocholine, which can be measured electrochemically^38,39^ and optically.^23^ Thiocholine can be readily measured through electrochemical oxidation on a gold^39^ or platinum^40^ working electrode or additional surface modifications with carbon black/Prussian blue nanoparticles,^38^ carbon nanotubes,^41,42^ or Fe_3_O_4_/Au nanocomposite^43^ to enhance the sensitivity of these methods at lower oxidation potentials. (iii) Combining ChE with choline oxidase (ChOx) to generate hydrogen peroxide, which can be measured through electrochemical^44^ or electrochemiluminescence^45^ approaches. The use of ChOx suffers from the need for an additional biological recognition element, leading to poor long-term stability, higher cost, and increased complexity; (2) detection of OP-AChE or OP-BChE adducts. A number of nanomaterial-based immunosensors, e.g., detection antibody labeled with Quantum dots, have been developed for sensitive electrochemical detection of OP-AChE and OP-BChE adducts,^46−48^ respectively. Although these measurements can indicate whether a person is exposed to OP pesticides, they cannot directly infer biological effects as ChE activity assays do; (3) simultaneously measuring ChE activity and total ChE (active and inhibited) by the combination of above two methods.^49−51^ This method has achieved a baseline (pre-exposure ChE)-free assessment of exposure to OP pesticides, which overcomes a limitation for ChE activity measurement methods. Although all of these methods are sensitive, have their own merits, and demonstrate great progress toward real world application, most of them still face significant challenges for POC testing due to either their complicated sample handling protocols, biofouling problems with real samples, or the lack of result tracking and data sharing.

To circumvent the limitations of gold standard methodologies and current biosensors for detecting OP pesticide exposure, we present the development of a highly stable, reagentless, low-cost, quantitative, and portable smartphone/resistive nanosensor platform for biomonitoring of exposure to OP pesticides. This platform integrates sample treatment components with the nanosensor, enabling the detection of OP pesticide exposure through temporal AChE/BChE measurements in finger-stick whole blood samples, without any external sample preprocessing. The novelty of this technology is the integration of a smartphone and Bluetooth resistance meter with the nanosensor consisting of a chitosan/multiwalled carbon nanotube/polyaniline nanofiber (CS/MWCNT/PAnNF) nanocomposite film atop a gold interdigitated electrode (AuIDE) and the unique transport properties of PAnNFs for the measuring resistance change of the nanosensor as a result of AChE or BChE’s hydrolysis of their respective substrate to generate protons doping the PAnNFs. As such, the change in resistance of the nanosensor is directly proportional to the activity of cholinesterase enzymes, which can be used for pesticide exposure assessment. Moreover, the mobile application is capable of rapidly processing, displaying, and storing AChE/BChE measurements, which, when paired with the simplicity and ease of testing (∼10 min for results), facilitates accurate baseline AChE/BChE activity determination through temporal measurements in comparison to aforementioned clinical methods. Under this approach, we demonstrate the capabilities of the nanosensor for both AChE and BChE measurements under physiologically relevant ranges with exceptional reproducibility and sensitivity. This nanosensing platform not only quantifies acute exposure to OP pesticides but also holds promise for evaluating chronic low-level exposures among farmworkers and the wider population.

## Materials and Methods

### Materials and Other Related Information

The readers are referred to the Supplementary Materials (S) for details: S1 for Reagents and materials, S2 for Instrumentation, S3 for Synthesis of multiwalled carbon nanotube (MWCNT)/polyaniline nanofiber (PAnNF) nanomaterials,^32,52^S4 for preparing heat-denatured whole blood samples for calibration curve development, S5 for preparation of *in vitro* spiked whole blood samples, S6 for details pertaining to validation of nanosensor with standard radiometric method, and S7 for details pertaining to validation of the nanosensor with standard Ellman’s method. Here, we present our sensor design and fabrication, app development, details on the field-testing study, and methodology for carrying out measurements in whole blood samples.

### Preparation of the CS/MWCNT/PAnNF-Modified Gold Interdigitated Electrode (AuIDE) Nanosensor

Preparation of the nanosensor and preloaded reagent pads are performed in a similar manner to our previous work,^32^ with some modification. The biosensor comprises four major components: a transducer AuIDE modified with CS/MWCNT/PAnNF, an outer pretreatment pad (outer pad) consisting of a glass fiber (GF) pad (outer Ø 4 mm and inner Ø 3 mm) preloaded with anti-interference reagents, e.g., AChE-specific inhibitor, BW284c51, and/or MgCl_2_ + CaCl_2_, and an inner signal generation pad (inner pad) consisting of a preloaded GF pad (Ø 2.5 mm) with an acetylcholine (ACh) or butyrylcholine (BCh) substrate.

Prior to fabrication of the biosensor, AuIDEs were cleaned as follows. First, AuIDEs were chemically polished by placing 50 μL of piranha solution consisting of 4 parts 98% H_2_SO_4_ and 1 part 30% H_2_O_2_ onto the AuIDE surface for 5 min. After chemical polishing, devices were rinsed with deionized (DI) water for 1 min and subsequently sonicated in acetone and DI water for 15 min each and dried *in vacuo*. To confine the sensing area, a parafilm template with a Ø 3 mm hole, made using a biopsy punch, was tightly attached to the AuIDE. After attaching the parafilm template, 10 μL of 0.0002% Tween-20 was placed on the exposed sensing surface for 15 min and subsequently rinsed with DI water for 1 min and dried *in vacuo* overnight.

To prepare MWCNT/PAnNF under the correct dedoping extent prior to drop-casting, we utilized a washing approach. Each wash treatment removed some protons from the PAnNFs. Specifically, 18 mg/mL 1.0 wt % MWCNT/PAnNF stock dispersion (fully doped in 1.0 M HCl) was centrifuged, and the supernatant was removed and replaced with water; this is a single wash. To achieve the desired dedoping extent and concentration, the MWCNT/PAnNF stock was washed 4 times and then diluted in water to the desired concentration. Next, the partially dedoped MWCNT/PAnNF suspension was lightly sonicated for 1 min and centrifuged for 3 s (to prevent casting aggregates onto the surface), and 4 μL of the MWCNT/PAnNF suspension was drop-casted onto the exposed sensing surface and allowed to air-dry. Following that, a protective layer consisting of 2 μL of CS was placed directly atop the nanocomposite transducer and was dried/stored *in vacuo* overnight at room temperature. Finally, the parafilm template was removed to reveal a CS/MWCNT/PAnNF-coated AuIDE and stored *in vacuo* before use.

To prepare the anti-interference outer pads, a GF membrane was cut into a ring, using a biopsy punch, with inner and outer Ø of 3 and 4 mm, respectively. For the measurement of whole blood AChE, outer pads are loaded with a 3 μL 1:1 molar ratio of MgCl_2_ + CaCl_2_ in DI water. For measurement of whole blood BChE, the outer pads are loaded with the same MgCl_2_ + CaCl_2_ concentration as the AChE outer pad plus AChE-specific inhibitor, BW284c51. After preloading the reagents, these outer pads were dried and stored *in vacuo* at room temperature. To prepare the inner pad, a GF membrane was cut into a circle using a biopsy punch with Ø 2.5 mm and 3 μL of ACh or BCh substrate was dispensed onto the circle GF pad and dried *in vacuo* at room temperature. Inner pads were stable for at least 6 months (data not shown), while the outer pads were stable for much longer time, e.g., 1 year, under dry conditions.

### Mobile App for Data Processing and Display

The mobile application developed for resistive nanosensor data processing and result display is a significantly enhanced version of that provided in our previous work.^32^ The mobile application was developed in Android Studio using JAVA and is capable of resistive signal preprocessing, peak identification (i.e., maximum resistance, addition of signal generation pad), data normalization (i.e., dividing all resistance data by the maximum resistance), and signal alignment (i.e., aligned response curves at the maximum resistance, start of generating signals). Furthermore, the application enables determination of AChE/BChE activity using experimentally derived calibration curves, displays results, illustrates temporal changes in AChE/BChE activity, collects user metadata (e.g., recently worked on crops, recently used brands of pesticides, health status, location, etc.), supports user account creation, and facilitates biosensor data transmission to a preliminary cloud/web-based platform (not discussed herein) and data storage and management.

### Field Testing

The experimental protocols for field testing were approved by the Institutional Review Board (IRB) of the Wake Forest University School of Medicine (no. IRB00072434) on March 14, 2021. Nanosensor field testing was conducted in August and September of 2021 with an initial recruitment of 25 farmworkers in Cumberland County, North Carolina, facilitated by our collaborators at Wake Forest University School of Medicine and North Carolina Farmworkers Project. There were 30 days between the two recruitments. Of the initial recruitment, 22 participated in both the first and the second field testings, 2 participated in only the first field testing, and 1 did not participate in either. The study involved questionnaires to collect metadata followed by blood sample collection using a finger-stick and micro capillary blood collection tube with lithium heparin to prevent coagulation. Each whole blood sample was split: half were immediately frozen using dry ice, and shipped for validation using the standard radiometric method, described in Section 6 in the Supporting Information. The other half of the sample was used for nanosensor measurements. A pipet was used to take 2 μL of whole blood for each ChE measurement using the nanosensor onsite (AChE and BChE, total 4 μL of whole blood). The data were received, analyzed, and displayed by the mobile application. The remaining half of the whole blood samples was sent to the lab with dry ice for additional nanosensor reproducibility testing. This includes mostly duplicates and up to six replicates per sample, totaling ∼300 tests. This study aimed to evaluate the nanosensor’s effectiveness with various samples in the field for ChE measurements, critical for POC assessment of farmworker exposure to OP pesticides.

### Sample Testing Protocol

The sensing platform consists of a nanosensor attached to an adapter (Figure S2A), which is connected to a Bluetooth multimeter (Figure S2D), and a mobile app (Figure S2E). The procedure for determining AChE and BChE activity and subsequently OP pesticide exposure is simple. First, a user-specific baseline of AChE and BChE activity is established through multiple testings. These ChE measurements are performed as follows: 15 μL of DI water and 2 μL of finger-stick whole blood are placed atop the sensing surface (Figure S2A). Next, an anti-interference outer pad is placed onto the sensing surface for 3 min (Figure S2B), containing anti-interference reagents dependent on the ChE type. The anti-interference reagents rapidly dissolve in the solution and inhibit whole blood AChE (for BChE measurements) and/or aid in neutralizing the blood bicarbonate buffer system. After incubation of the sample with the anti-interference outer pad, a signal generation inner pad containing either ACh or BCh substrate is placed atop the sensing surface (Figure S2C). ACh or BCh rapidly dissolves in the diluted whole blood and is hydrolyzed by AChE or BChE, respectively, generating a response of the resistance change over time. Response data are collected for 5 min after the start of the signal generation and uploaded to the mobile application for data processing and visualization (Figure S2E). As such, the conductance change is directly proportional to AChE or BChE activity in a whole blood sample, which is quantified using a calibration curve. Sequential ChE measurements are obtained in a consistent manner and are stored within the mobile application. These ChE measurements are then used to determine the baseline (i.e., maximum AChE/BChE activity, uninhibited) and compared with future activity levels to determine exposure or recovery from OP pesticide poisoning. The enzyme inhibition is calculated using the following equation:
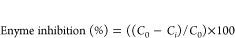


Here, *C*_0_ is pre-exposure enzyme activity. *C_i_* is postexposure enzyme activity.

## Results and Discussion

### Principle of the Method

**Figure 1:**
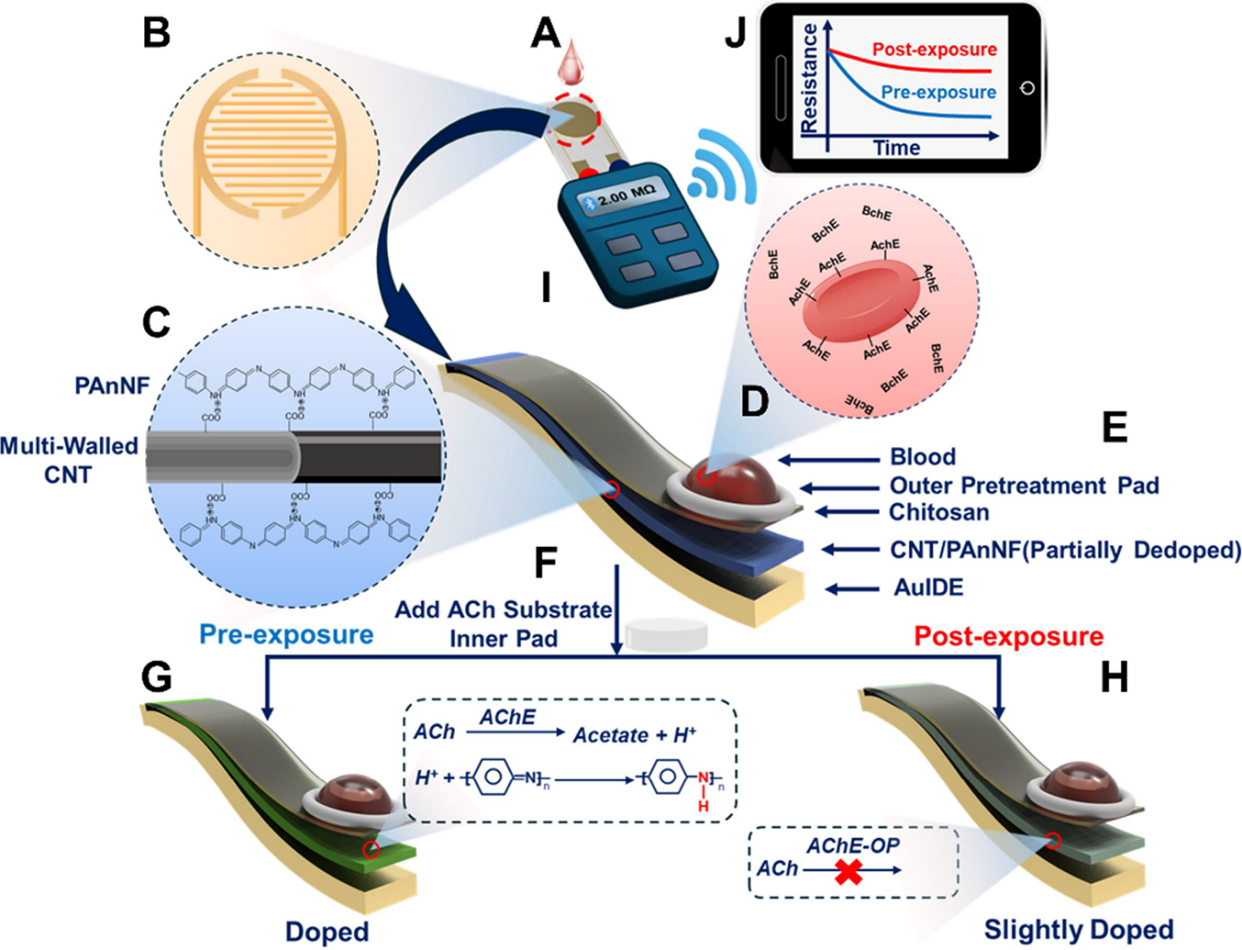
Schematic of the CS/MWCNT/PAnNF smartphone-nanosensor for the detection of human exposure to OP pesticides using AChE as a model. (A) Application of a finger-stick whole blood sample to (B) AuIDE coated with (C) CS/MWCNT/PAnNF/nanocomposite. (D) Whole blood sample containing AChE and BChE interacts with the nanosensor and is pretreated with a (E) GF outer pretreatment pad. (F) After pretreatment (ChE-specific), an ACh substrate loaded GF pad is placed onto the sensing surface and rapidly dissolves. (G) Prior to OP pesticide exposure, AChE is fully active and rapidly hydrolyzes dissolved ACh to produce a large quantity of protons, which heavily dopes the CS/MWCNT/PAnNF film. (H) However, upon exposure to OP pesticides, AChE activity is depreciated, which reduces the amount of protons generated, resulting in a slightly doped CS/MWCNT/PAnNF film. (I) CS/MWCNT/PAnNFs-coated AuIDE is connected to a multimeter, which measures the resistance change overtime and transmits resistive data to a smartphone through Bluetooth. (J) Smartphone app processes resistive data and displays changes in AChE/BChE activity for pre- (blue line) and postexposure (red line). Pesticide-induced inhibition of AChE and/or BChE results in reduced proton generation and thus a reduced resistance change.

The principle of the device is based on AChE or BChE-facilitated hydrolysis of their respective substrate, ACh or BCh, and the unique transport properties of PAnNFs^53,54^ (i.e., proton doping (i.e., H^+^ adding to imine nitrogen in the PAnNF backbone) greatly increases the conductance of PAnNFs due to the formation of charge carriers (e.g., holes and delocalized π-electrons)), see Figure S4. Specifically, a AuIDE (Figure 1A,B) is coated with a MWCNT/partially dedoped PAnNF (Figure 1C) nanocomposite, whose transport properties are affected by changes in local pH (generation of H^+^ or OH^–^).

Within a finger-stick whole blood sample, both AChE and BChE are present (Figure 1D) and susceptible to inhibition upon exposure to OP pesticides. The enzymatic hydrolysis of their respective substrate results in the formation of choline and either acetic acid or butyric acid, respectively, at a rate of 25,000 molecules of substrate per second. Notably, AChE exhibits higher enzymatic activity and is capable of cohydrolyzing BCh at a fast rate. Thus, to ensure accurate BChE measurements, AChE is inhibited using the AChE-specific inhibitor, BW284c51, within the outer pretreatment pad (Figure 1E). On the other hand, BChE hydrolyzes ACh at a much slower rate, particularly at low ACh concentrations,^55^ and has much lower activity in comparison to AChE (the catalytic rate constants (*k*_cat_) for AChE and BChE being 1.6 × 10^4^ and 0.1 × 10^4^ s^–1^, respectively^56,57^). Due to this difference, inhibiting BChE is unnecessary for AChE measurements.

Figure 1 schematically illustrates the sensing principle of the smartphone-nanosensor for quantifying the AChE activity. In consideration of a two time-point test, pre- and postexposure, a user’s baseline (pre-exposure enzyme activity) shows uninhibited AChE activity levels. Specifically, ACh preloaded in the inner pad (Figure 1F) is rapidly hydrolyzed by AChE resulting in significant generation and local accumulation of protons, which rapidly dopes PAnNFs in the CS/MWCNT/PAnNF nanocomposite (Figure 1G), thereby increasing conductance across the AuIDE (Figure 1J, blue line). The change in conductance over time is measured using a Bluetooth multimeter (Figure 1I) and is transmitted to the smartphone mobile app for resistive data processing and display (Figure 1J). However, exposure to OP pesticides leads to depreciated ChE activity levels, reducing proton generation (Figure 1H) and subsequently lowering the conductance change across the AuIDE (Figure 1J, red line). As such, the change in conductance across the AuIDE is directly proportional to ChE activity in a whole blood sample. Thus, through POC temporal measurements of AChE and BChE activity levels in whole blood, determination of OP pesticide exposure or recovery becomes feasible and accessible using this nanosensor platform.

### Characterization of MWCNT/PAnNF Nanocomposite

Characterization of the hybrid MWCNT/PAnNF nanocomposite is available in our previously published work for the assessment of pesticide in food and water.^32^ Briefly, transmission electron microscopy (TEM) confirmed the presence of nanofiber morphologies with ∼80 nm diameters in the 1.0 wt % MWCNT/PAnNF dispersion (Figure S3A). Ultraviolet–visible spectroscopy (UV–vis) confirmed the presence of emeraldine salt PAnNFs; furthermore, the absorbance intensity at around 350 nm was utilized for determining the concentration of the MWCNT/PAnNF suspension (Figure S3B). This concentration adjustment ensured precise tuning of the film’s thickness prior to drop-casting onto the AuIDE, ensuring the consistency and reproducibility of the nanocomposite films within the confined sensing area. Furthermore, the ratio of the π–π* (350 nm) to polaron−π* (430 nm) transition was used to estimate the extent of PAnNF doping,^58^ in addition to the dispersion pH. However, initial dry resistance of the CS/MWCNT/PAnNF film predominantly served as a quality control measure to validate the correct doping/dedoping extent.

### Optimization of Experimental Parameters

Nanosensor optimization parameters (e.g., MWCNT content, MWCNT/PAnNF thickness, CS thickness, ACh and BCh concentration, whole blood volume, MgCl_2_ + CaCl_2_ pretreatment concentration, BW284c51 AChE inhibitor concentration, and sample pretreatment incubation period) were performed using fresh whole blood samples (Zenbio, Inc.) to ensure ideal nanosensor parameters for real sample testing. Furthermore, a blood buffer system such as carbonic acid/bicarbonate ions can neutralize protons generated from ACh/BCh hydrolysis and rapidly strip protons from the PAnNFs. Thus, MgCl_2_ + CaCl_2_ is used to pretreat blood to destroy the buffer system by precipitation of carbonate anions with Mg^2+^ and Ca^2+^. To further decrease the effects of the blood matrix on the nanosensor, only 2 μL of whole blood sample is used. In general, all nanosensor measurements were performed with 15 μL of DI water and 2 μL of whole blood on the surface. Finally, the MWCNT/PAnNF film was dedoped to approximately 3 MΩ prior to the addition of the whole blood sample, which redopes the MWCNT/PAnNF to approximately 1.2 MΩ baseline resistance - (i.e., the resistance prior to adding the substrate pad to generate the signals).

For the development of the nanosensor aimed at measuring AChE and BChE as OP pesticide exposure biomarkers, the same 1.0% wt. MWCNT content as selected in our prior work was used.^32^ Specifically, MWCNTs were integrated into a hybrid MWCNT/PAnNF nanocomposite to enhance the stability and reproducibility of the PAnNFs. Previously, it was found that increasing the MWCNT content resulted in reduced nanosensor sensitivity but significantly improved stability and reproducibility of PAnNFs. As such, 1.0% wt. MWCNT was previously determined to be the optimal amount and is routinely used, providing sufficient sensitivity while retaining exceptional nanosensor reproducibility based on local pH changes.

**Figure 2:**
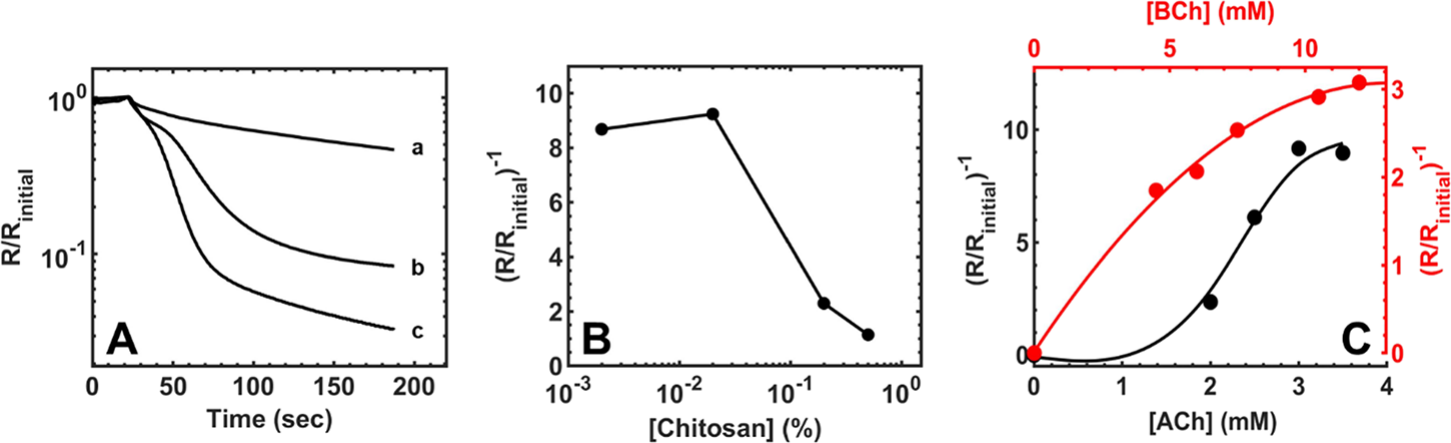
(A) Response of nanosensors with different thicknesses of MWCNT/PAnNFs from dilutions of stock MWCNT/PAnNF suspension (mg/mL): (a) 0.84, (b) 1.12, and (c) 1.68. Responses generated with 2.0 μL of 8.8 U/mL eel AChE in water and a 3.0 mM surface ACh concentration. (B) Response of nanosensors coated with different amounts of chitosan (%): 0.002, 0.02, 0.2, and 0.5 on the surface of the MWCNT/PAnNF film using a 2.0 μL whole blood sample and 15.0 μL of 3.0 mM ACh. (C) Response of nanosensors with different final surface concentrations of GF-loaded (black) ACh (mM): 0, 2.0, 2.5, 3.0, and 3.5; and different concentrations of GF-loaded (red) BCh (mM): 0, 4.5, 6.0, 7.5, 10.5, and 12.0, using a 2.0 μL whole blood sample and 15.0 μL of DI water.

Next, the effect of the MWCNT/PAnNF film thickness was investigated. Initially, UV absorbance at 350 nm was used to quantify the amount of MWCNT/PAnNFs in the dispersion prior to drop-casting. MWCNT/PAnNF film thickness was evaluated based on the following factors: dedoping rate, response time, and signal intensity (i.e., Ohm change). Figure 2A illustrates the responses of different MWCNT/PAnNF film thicknesses with 2 μL of 8.8 U/mL eel AChE and 15 μL of 3 mM ACh. The nanosensor’s response increased with increasing thickness of MWCNT/PAnNF, showing the highest response at 1.68 mg/mL MWCNT/PAnNF. However, this configuration suffered from poor analytical time as a result of the slow dedoping rate to reach baseline resistance (i.e., resistance prior to the addition of the substrate pad) due to an abundance of H^+^-doped sites and reduced initial response generation time due to hindered diffusion capabilities. Thus, the 1.12 mg/mL (Abs 0.65 at 350 nm) MWCNT/PAnNF dispersion was selected for future studies, in light of its sufficient dedoping rate, fast response time, and large Ohm change.

To mitigate nonspecific binding from proteins and metabolites present within a whole blood sample onto the sensing surface, CS was employed as a protective membrane. CS is a widely used biopolymer with an abundance of functional groups that can mitigate nonspecific binding of the blood matrix onto the MWCNT/PAnNF AuIDE transducer,^59^ thus reducing their effects on nanosensor performance. Under optimized MWCNT/PAnNF conditions, CS/MWCNT/PAnNF-modified AuIDEs were evaluated with 2 μL of varying concentrations of CS (0.002–0.5%) using 2 μL of whole blood samples and an ACh-preloaded inner pad (Figure 2B). The effect of the CS layer was optimized with various considerations, including nonspecific binding of the whole blood matrix to MWCNT/PAnNF, diffusion of substrates and protons to MWCNT/PAnNF, and the dedoping rate of PAnNFs. As seen in Figure 2B, the signal intensity of the CS/MWCNT/PAnNF-modified AuIDE decreased with increasing CS concentrations. The observed relationship is expected due to reduced diffusion capabilities with increasing CS film thickness. Lastly, sufficient Ohm changes were observed for all CS concentrations below 0.02%. Therefore, 2 μL of 0.02% CS was selected as the optimal concentration for the CS/MWCNT/PAnNF-modified nanosensor because of its ability to reduce nonspecific binding of the blood matrix and retention of diffusion capabilities to the MWCNT/PAnNF transducing layer.

Using optimal CS/MWCNT/PAnNF film parameters, substrates were optimized with 3 μL of ACh or BCh dried on an inner pad with Ø 2.5 mm, to achieve final surface concentrations ranging from 0 to 3.5 mM and 0 to 12 mM (17 μL of surface volume, 15 μL of water + 2 μL of whole blood), respectively. ACh and BCh were optimized by using 2 μL of the same whole blood sample. As demonstrated in Figure 2C, the signal intensity of the nanosensor is linearly proportional to ACh and BCh concentrations, until saturation at 3 and 10.5 mM, respectively. Therefore, surface concentrations of 3 mM ACh and 10.5 mM BCh were selected for all future experiments.

Given the complex composition of whole blood (e.g., ions, proteins, metabolites, etc.), the effect of whole blood volume on nanosensor performance was investigated under the following criteria: (i) baseline resistance of a nanosensor, (ii) the dedoping rate of the nanosensor during pretreatment; and (iii) signal generation of the nanosensor. Here, the baseline resistance is critical for determining the signal intensity and reproducibility of the nanosensor. The effect of baseline resistance on signal intensity was first investigated in water using 0.1323 U eel AChE, at high and low baseline resistances (Figure S5). It was found that a higher baseline resistance led to increased signal intensity and, thus, improved sensitivity. This was attributed to the fact that an increased abundance of dedoped PAnNFs (i.e., high baseline resistance, increased doping sites) allows more protons to easily dope the PAnNFs, thus generating high signals.

**Figure 3:**
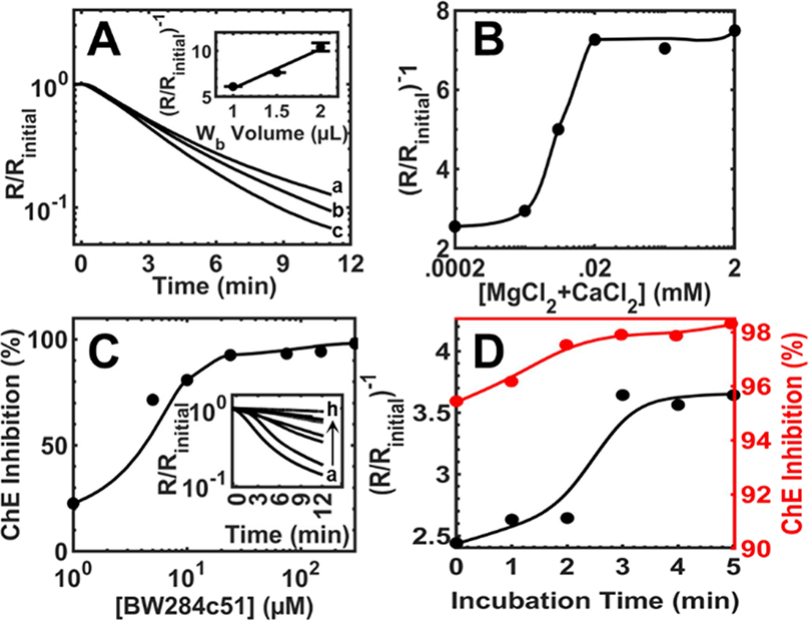
(A) Response of the nanosensor under optimal conditions with different whole blood sample volumes (μL): (a) 1.0, (b) 1.5, and (c) 2.0. Responses were generated with 15 μL of DI water and 3 mM surface ACh (GF loaded). (B) Response of the nanosensor under different surface concentrations of GF-loaded MgCl_2_ + CaCl_2_ (mM): 0.0002, 0.002, 0.006, 0.02, 0.2, and 2.0 with 2.0 μL of whole blood sample. Responses generated with 15 μL of DI water, 3.0 mM surface ACh (GF loaded), and 5.0 min of incubation. (C) Inhibition of AChE in 2.0 μL of whole blood by different surface concentrations of GF-loaded BW284c51 (μM): (a) 0.0, (b) 1.0, (c) 5.0, (d) 10.0, (e) 25.0, (f) 75.0, (g) 150.0, and (h) 300.0. Responses generated with 15 μL of DI water, 3.0 mM surface ACh (GF loaded), and 5 min of incubation. (D) Optimization of pretreatment time for 2.0 μL of whole blood with (black) 0.2 mM MgCl_2_ + CaCl_2_ and (red) 150.0 μM BW284c51 inhibitor and 0.2 mM MgCl_2_ + CaCl_2_ at periods of (minutes): 0.0, 1.0, 2.0, 3.0, 4.0, and 5.0. Responses generated with 15.0 μL of DI water and 3.0 mM surface ACh (GF loaded).

Next, studies on the effect of whole blood volume on nanosensor performance revealed the following critical findings: (i) It was observed that adding whole blood to the sensor surface resulted in a notable decrease of baseline resistance in comparison to that in water (data not shown). Moreover, the addition of whole blood volumes greater than 1 μL stabilized the nanosensor’s baseline resistance prior to the addition of the substrate pad, which coincidentally enhanced the reproducibility tremendously. Furthermore, by reaching this steady-state baseline resistance during sample incubation, nanosensor response generation is completely dependent on the hydrolysis of the ACh/BCh substrate by AChE/BChE. This stabilization of baseline resistance was ascribed to the moderate conductivity of whole blood due to the presence of various salts such as sodium and potassium ions. To confirm this, 2 μL of 140 mM NaCl, which is the typical concentration of salt in whole blood, was added to the surface of the nanosensor and was found to sharply drop the baseline resistance and then stabilize the resistance in a similar fashion to whole blood (Figure S6). (ii) Signal intensity of the nanosensor was found to linearly increase with increasing whole blood volumes from 1.0 to 2.0 μL (Figure 3A), reflecting a proportional increase in AChE quantity on the nanosensor surface. Based on these findings, 2.0 μL of whole blood was determined as the optimal sample volume for the nanosensor, ensuring high baseline resistance, maximum signal intensity, and highly reproducible measurements.

### Interference Effects

Aside from nonspecific binding of the blood matrix to the CS/MWCNT/PAnNF transducer, interferences from the following were considered: (i) the blood carbonic acid/bicarbonate buffer system due to the consumption of protons generated from the ChE-facilitated hydrolysis of ACh/BCh. This consumption of protons would affect the local pH dynamics on the sensor surface, reducing the conductance changes observed during measurements; (ii) presence of AChE for BChE measurements, due to high catalytic activity of AChE and its capability to cohydrolyze BCh at a rapid rate. Importantly, it was concluded that the reverse scenario for interference was negligible, since the activity of BChE is significantly lower than AChE and ACh hydrolysis by BChE is significantly slower at lower ACh concentrations.^55^ This conclusion was experimentally supported (Figure S7), where no significant response was generated in plasma (highest concentration of BChE) in the presence of 3 mM ACh.

To mitigate the effects of the blood carbonic acid/bicarbonate buffer on sensing performance, calcium (Ca^2+^) and magnesium (Mg^2+^) cations were preloaded into the outer pad with outer Ø 4 mm and inner Ø 3 mm (similar to ACh, cation concentrations are provided as surface concentrations). These cations were selected to precipitate carbonate (CO_3_^2–^) anions in blood, thereby destroying the carbonic acid/bicarbonate buffer capacity. The concentrations of Ca^2+^ and Mg^2+^ cations were optimized, and results are provided in Figure 3B. As can be seen, increasing the concentrations of these cations led to a corresponding increase in signal intensity of nanosensors by a factor of 3, plateauing at 20 μM. Additionally, the effects of Ca^2+^ and Mg^2+^ cations on a control signal, without the presence of ACh or BCh were investigated. It was found that no significant change in resistance (i.e., no signal generation) occurred upon the addition of cations to the sensing surface, without the presence of a signal generation inner pad. To ensure that blood buffer interference is minimized in all cases, 200 μM (surface concentration, 1:1 molar ratio, CaCl_2_:MgCl_2_) cations were selected for further studies to minimize carbonic acid/bicarbonate buffer interference consistently across varying samples. In addition, protons released from destroying the carbonic acid/bicarbonate buffer system may dope the PAnNF and contribute to stabilizing the baseline resistance before adding an inner pad to generate signals.

To address the issue of BCh hydrolysis by AChE during whole blood BChE activity measurements, the AChE-specific inhibitor BW284c51 was utilized. BW284c51 is a well-known AChE-specific inhibitor commonly used for treatment of Alzheimer’s^60^ and BChE activity assays using Ellman’s method.^61^ The AChE-specific inhibitor was predried on the same outer pad containing the Ca^2+^/Mg^2+^ cations (similar to prior, inhibitor concentrations are provided as surface concentrations). Figure 3C illustrates that as the concentration of the BW284c51 inhibitor increases, inhibition of AChE activity also increases. Complete inhibition of AChE activity was achieved at 150 μM after a 5 min incubation period. As such, 150 μM BW284c51 was selected for all future measurements of whole blood BChE activity, ensuring minimal interference from AChE. With optimized pretreatment concentrations (i.e., Ca^2+^/Mg^2+^ and BW284c51), the incubation period for the pretreatments was optimized and is provided in Figure 3D. It was found that a 3 min incubation period was sufficient for the cations to effectively disrupt the carbonic acid/bicarbonate buffer capacity and for BW284c51 to inhibit all AChE activity within a whole blood sample. As such, 3 min was selected as the ideal incubation period for all pretreatments of blood samples on the sensor surface.

### Analytical Performance of the Nanosensor

**Figure 4:**
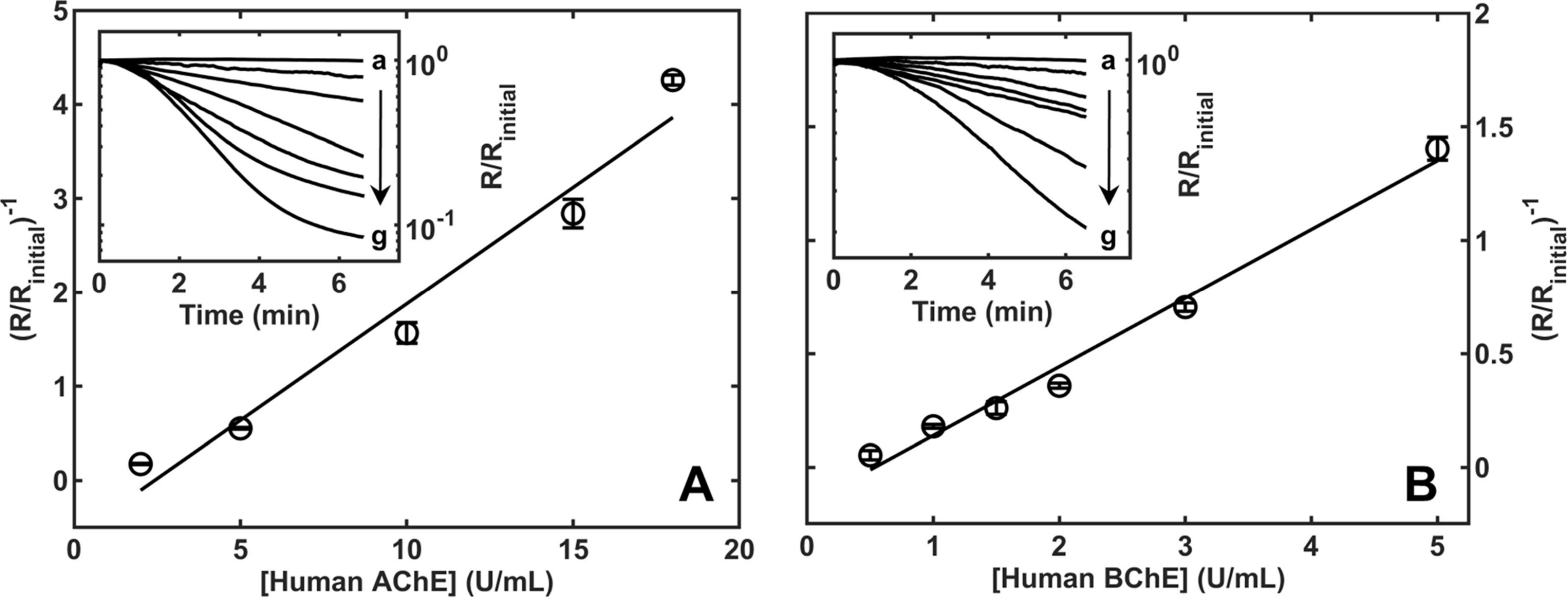
(A) Calibration curve of the nanosensor for measuring AChE in blood samples under optimal conditions with all ChE-specific pretreatments and substrates loaded on GF pads. Heat-denatured whole blood spiked with different concentrations of human AChE (units per milliliter) was (a) 0.0, (b) 2.0, (c) 5.0, (d) 10.0, (e) 15.0, (f) 18.0, and (g) 20.0. (B) Calibration curve of the nanosensor for measuring BChE in whole blood under optimal conditions. Heat-denatured whole blood spiked with different concentrations of BChE (units per milliliter): (a) 0.0, (b) 0.5, (c) 1.0, (d) 1.5, (e) 2.0, (f) 3.0, and (g) 5.0.

The analytical performance of the nanosensor for measuring AChE and BChE activity in whole blood samples was evaluated using heat-denatured whole blood spiked with ChE and is prepared as outlined in Section 4 of the Supporting Information. Using heat-denatured whole blood, the analytical performance can be assessed under exact testing conditions and provides a highly accurate calibration curve for quantification under physiologically relevant ranges. This varies tremendously from other reported biosensors where analytical performance is provided under ideal conditions (i.e., water or buffer). The calibration curve of normalized resistance changes vs AChE or BChE activity is provided in Figure 4. Six replicates of a blank sample (control, heat-denatured blood, no ChE) and each concentration of AChE or BChE were evaluated by the nanosensor. According to Figure 4, the calibration curve equation for AChE (Figure 4A) is *y* = 0.24786*x* – 0.59987 with the *R*^2^ value at 0.98, detection limit at 0.11 U/mL (based on 3σ/slope), and a dynamic range between 2.0 and 18.0 U/mL. The calibration curve equation for BChE (Figure 4B) is *y* = 0.30262*x* – 0.16209 with the *R*^2^ value at 0.99, detection limit at 0.093 U/mL, and a dynamic range between 0.5 and 5.0 U/mL. The nanosensor is highly reproducible with RSD for AChE and BChE calibration curve data at 2.07 and 1.15%, respectively. These calibration curves were integrated into the mobile app for measuring AChE and BChE activity in whole blood samples. The total analysis time for each measurement, including the pretreatment period, is approximately 8 min.

Comparison with biosensors reported over the last two decades (Table S1) revealed that this work provided superior analytical performance across all parameters. Specifically, it enables measurement of both ChE biomarkers using a whole blood sample without external sample preparation such as centrifugation or pretreatment. Furthermore, the nanosensor demonstrated a short analytical time, exceptional reproducibility, and coverage of the biologically relevant range under real sample conditions, highlighting its suitability for applications in-field settings.

**Figure 5:**
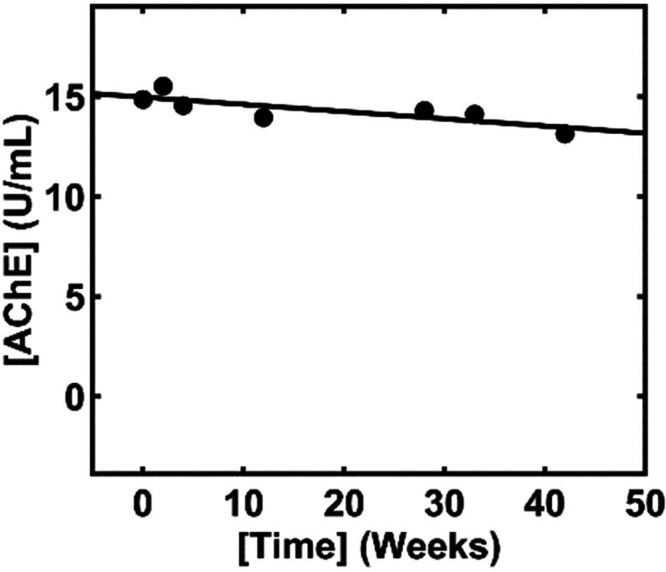
Long-term stability of CS/MWCNT/PAnNF-modified AuIDE nanosensors under optimal conditions for AChE measurements in whole blood at various time points (weeks): 0.0, 2.0, 4.0, 12.0, 28.0, 33.0, and 42.0. CS/MWCNT/PAnNF-modified AuIDE nanosensors were stored under constant dry vacuum conditions.

The long-term stability of the nanosensor for determining AChE activity in whole blood was assessed over the course of one year using a whole blood sample stored at −20 °C. A batch of nanosensors was prepared as previously described and stored in a vacuum desiccator at room temperature prior to testing. A baseline measurement (day 0) was established to determine whether there is any loss in performance during subsequent testing. The results from the long-term stability study are provided in Figure 5. As shown in the figure, nanosensors within the same batch exhibited exceptional stability for determining whole blood AChE activity over the course of one year. The RSD among all measurements within the batch was found to be 5.2%, and a decrease of 11.6% was determined between week 0 and week 42, indicating sufficient long-term stability. The high stability is attributed to the absence of biological recognition elements (e.g., enzymes or antibodies) and the inherently high stability of the CS/MWCNT/PAnNF nanocomposite film under dry conditions. It is important to note that while the nanosensor demonstrates excellent stability under dry conditions, exposure to high humidity levels would degrade the film over time due to gradual stripping of protons. As such, under controlled dry conditions, this platform demonstrates exceptional long-term stability for POC settings in comparison to other reported biosensors for the determination of human exposure to pesticides.

### Assessment of the Nanosensor with *In Vitro* Spiked Whole Blood Samples

**Figure 6:**
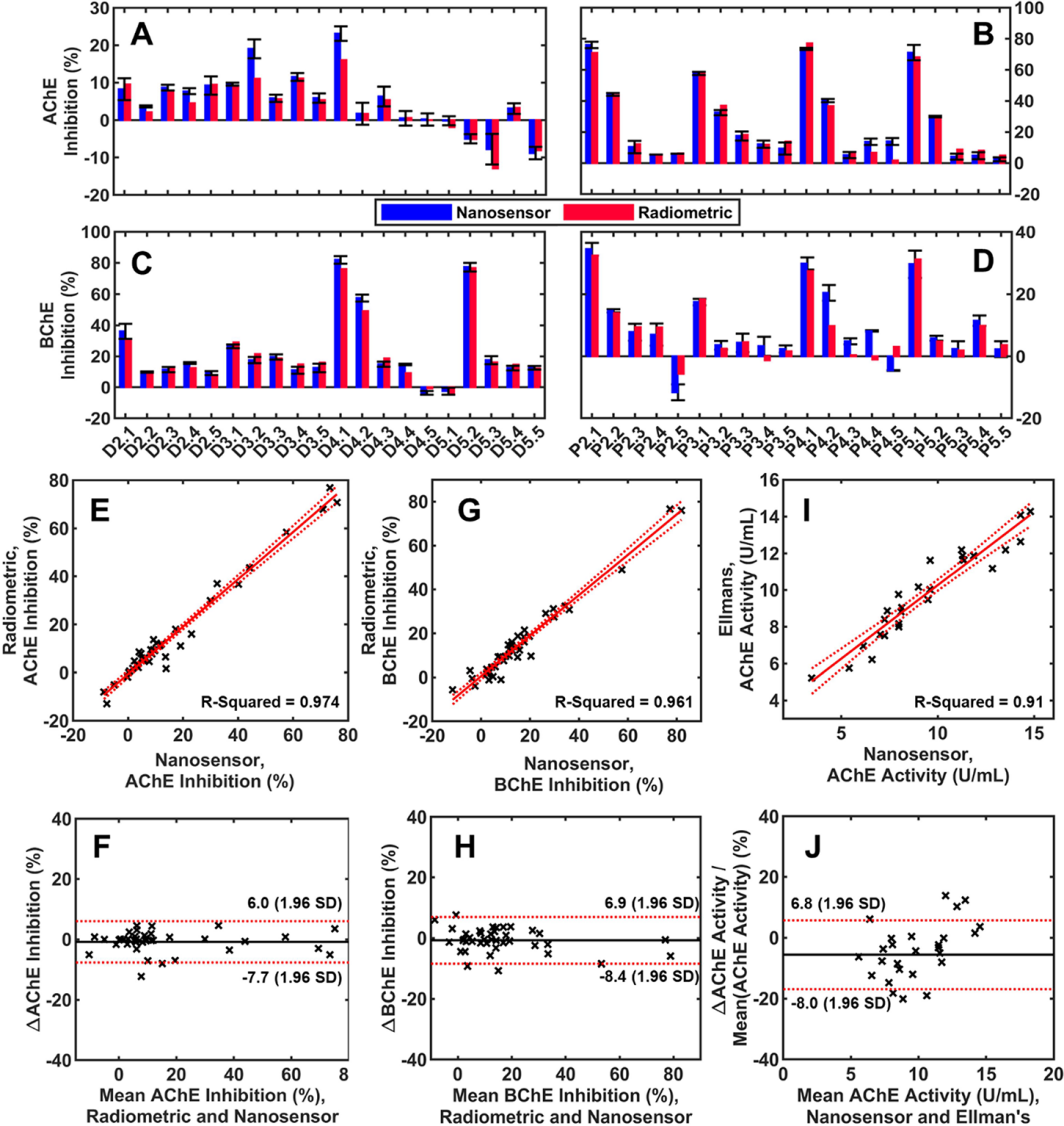
Comparison of AChE and BChE inhibition measurements from the nanosensor-smartphone platform and radiometric method using four whole blood samples spiked with five different concentrations of Diazinon-Oxon or Paraoxon-methyl. (A) AChE inhibition by Diazinon-Oxon, (B) AChE inhibition by Paraoxon-methyl, (C) BChE inhibition by Diazinon-Oxon, and (D) BChE inhibition by Paraoxon-methyl. (E) Linear model for AChE inhibition between the nanosensor and radiometric method. (F) Bland–Altman plot for AChE inhibition between the nanosensor and radiometric method. (G) Linear model for BChE inhibition between the nanosensor and radiometric method. (H) Bland–Altman plot for BChE inhibition between the nanosensor and radiometric method. (I) Linear model for AChE activity between the nanosensor and Ellman’s method with 28 samples and (J) Bland–Altman plot for AChE activity between the nanosensor and Ellman’s method.

Pesticide-spiked whole blood samples were prepared *in vitro* as outlined in Section 5 in the Supporting Information for validating the capabilities of the biosensor for whole blood AChE and BChE measurements. Under optimal nanosensor conditions, whole blood AChE and BChE for each sample were measured using 2.0 μL of whole blood for each biomarker, respectively, and each sample was tested in triplicates (total ∼300 testing). The reproducibility of the nanosensor for measuring *in vitro* AChE and BChE-spiked samples resulted in an RSD of 2.75%. In parallel, whole blood AChE and BChE for the other half of the samples were analyzed using a standard radiometric method. The results of enzyme inhibition for each Diazinon-Oxon (Figure 6A,C) or Paraoxon-methyl (Figure 6B,D) spiked whole blood samples from the two methods are summarized in Figure 6. A linear regression model was built to initially understand the consistency between the nanosensor and standard radiometric method. Briefly, AChE (Figure 6E) and BChE (Figure 6G) inhibition results from the two methods were plotted against each other, where each point represents sample inhibition measured by the nanosensor and radiometric method. High consistency between the nanosensor and radiometric measurements was observed; the linear correlation results between the two methods for AChE (Figure 6E, *R*^2^ = 0.974) and BChE (Figure 6G, *R*^2^ = 0.961) are both greater than 0.95. Furthermore, the linear model equations for AChE and BChE were found to be *y* = 0.9845*x* – 0.5965 and *y* = 0.9214*x* + 0.5262, respectively. From these equations, it is clear that the two methods are consistent, given that the slopes are close to 1 and the *y*-intercepts are close to 0. Next, the consistency between the two methods was further evaluated using Bland–Altman plots (Figure 6F,H). The mean values of the difference in inhibition for AChE and BChE, between the two methods, were −0.84 and −0.74% with 95% confidence intervals (CI) of −7.7 to 6% and −8.4 to 6.9%, respectively.

Further validation was performed against Ellman’s method, utilizing 28 *in vitro* samples for AChE measurements (triplicates). The methodology for Ellman’s assay is outlined in Section 7 in the Supporting Information, along with the calibration curve used for quantification (Figure S1). Comparative analysis between Ellman’s method and the nanosensor is provided in terms of AChE activity (U/mL). A strong linear correlation (Figure 6I, *R*^2^ = 0.91) between the Nanosensor and Ellman’s method is observed for whole blood AChE measurements. However, the nanosensor underestimated cholinesterase results relative to Ellman’s method (*y* = 0.7994*x* + 2.264) given that the slope is significantly lower than 1. This was further observed in the Bland–Altman plot (Figure 6J), where the mean percent difference between measured AChE activities is −5.59 and the 95% confidence interval is −8 to 6.8%. Relatively high variation of AChE measurements between methods was primarily attributed to the intrinsic variation of Ellman’s method as demonstrated with an average RSD of 9.02 ± 7.16% (*n* = 28, triplicates, 84 total), in comparison to the nanosensor being 2.78% (*n* = 48, triplicates, 144 total), and is further illustrated with a boxplot provided in Figure S8. Furthermore, the high presence of interfering compounds (e.g., thiols, hemoglobin, etc.) and delayed processing of frozen whole blood samples likely further compounded the differences observed. Based on the comparative analysis against the standard radiometric and Ellman’s method, the nanosensor provides compelling evidence for potential application in POC analysis of OP pesticide exposure using cholinesterase measurements.

### Validation of the Nanosensor with Field-Testing Samples

**Figure 7:**
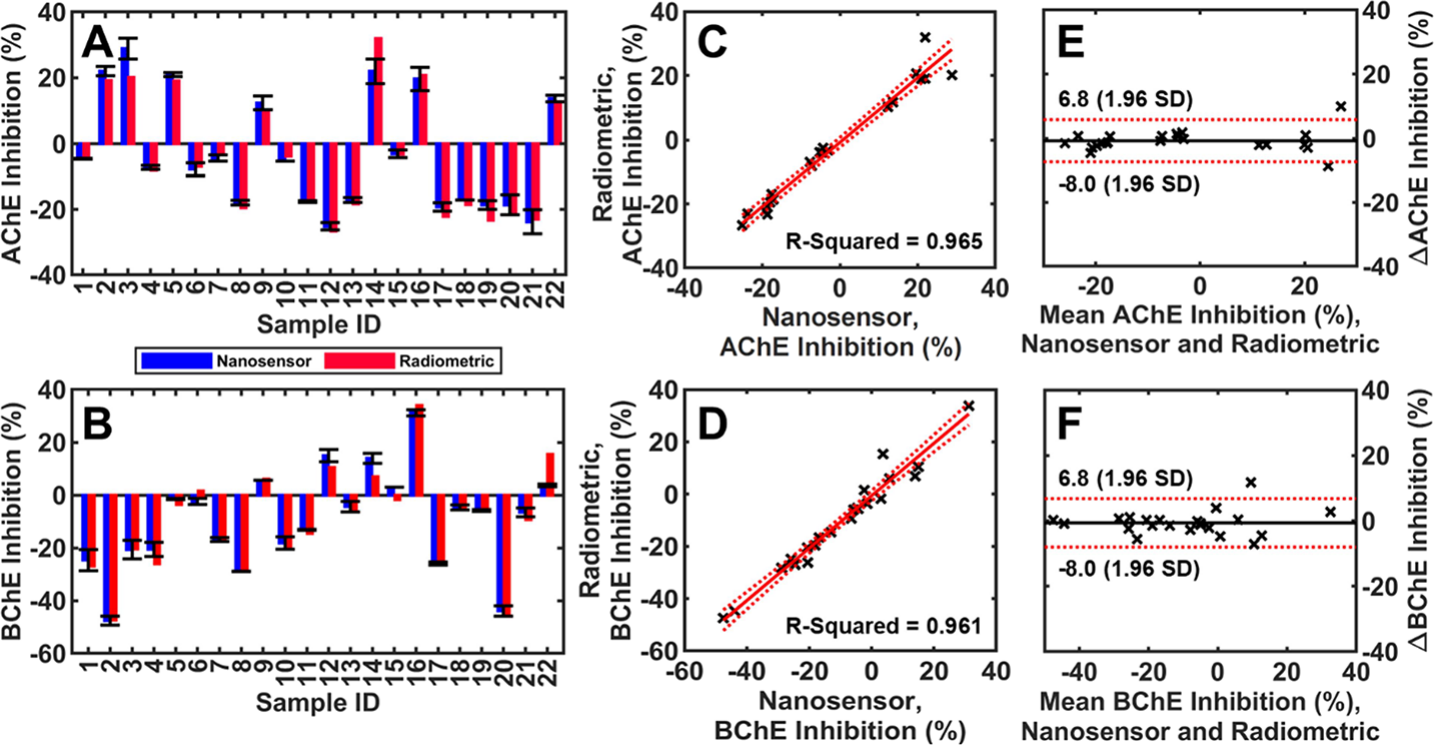
Bar plots and comparative analysis of each participant’s whole blood ChE activity changes during two recruitments measured by our nanosensor and radiometric method, respectively: (A) AChE changes and (B) BChE changes. (C) Linear model of each participant’s AChE changes obtained from our nanosensor and radiometric method. (D) Linear model of each participant’s BChE changes obtained from our nanosensor and radiometric method. Bland–Altman plots of the mean ChE inhibition vs the difference in ChE inhibition between the radiometric and nanosensor method for (E) AChE and (F) BChE inhibition measurements.

To demonstrate the potential application for onsite testing of exposure to OP pesticides, we further examined the nanosensor using finger-stick blood from farmworkers in Cumberland County, North Carolina. Out of the initial 25 participants recruited, 22 completed both rounds of sample collection, 2 completed only the first round, and 1 did not participate in either. Those that did not complete both testing rounds were excluded from the study presented. The details on how to collect the finger-stick blood samples and test the blood samples in the field are outlined in the Materials and Methods section. Briefly, 22 farmworker blood samples were evaluated using the nanosensors to measure the changes in AChE and BChE activity during the months of August and September 2021. In parallel, these blood samples were measured using the radiometric method, too. The results of AChE changes (Figure 7A) and BChE changes (Figure 7B) of farmworkers during the field-testing period from these two methods are summarized in Figure 7. The nanosensor demonstrated exceptional reproducibility for AChE and BChE (RSD = 2.55%, *n* = 22, triplicates and some sextuplicates, ∼300 total). A linear regression model and Bland–Altman plot were used to determine the agreement between the nanosensor and radiometric method for field testing. Linear regression analysis revealed exceptional consistency between the nanosensor and radiometric method. The *R*^2^-values of the linear models were 0.965 for AChE (Figure 7C, *y* = 1.004*x* – 0.7336) and 0.961 for BChE (Figure 7D, *y* = 1.003*x* – 0.5823). From these linear models, it is clear that there is a strong agreement between methods given that the slopes are close to 1 and the *y*-intercepts are close to 0. To further evaluate the consistency, Bland–Altman plots were generated, showing a mean difference of inhibition of −0.75% (−8.0 to 6.8%, 95% CI) for AChE (Figure 7E) and −0.62% (−8.0 to 6.8, 95% CI) for BChE (Figure 7F). As such, these results further demonstrate the capabilities of the nanosensing platform for in-field biomonitoring and assessment of OP pesticide exposure.

Briefly, changes in farmworker AChE and BChE activities were analyzed to infer potential exposure to OP pesticides or recovery from the exposure. Figure 7A,B indicates that Farmworker 16 (the number represents sample ID in Figure 7) had over subclinical exposure with >20% inhibition of both AChE and BChE. Farmworker 14 also showed over subclinical exposure with >20% inhibition of AChE but <20% inhibition of BChE. Farmworkers 9 and 22 showed subclinical exposures with <20% inhibition of both AChE and BChE. Farmworkers 2, 3, and 5 had >20% AChE inhibition. But Farmworkers 2 and 3 had >20% recovery in BChE; Farmworker 5 had less than 5% BChE recovery. The data indicate that farmworkers 2, 3, 5, 9, 14, 16, and 22 may have had long-term low-level exposures to OP pesticides. Farmworkers 9, 12, 14, 16, and 22 may have recent low-level exposures to OP Pesticides. Farmworkers 12 and 21 showed >20% recovery in AChE. Farmworkers 1, 2, 8, 17, and 20 showed >20% recoveries in BChE. Other farmworkers except 15 showed increasing cholinesterase (either AChE, or BChE, or both) during the month, indicating that these participants enzyme activity was restored from previous subclinical low-level exposures to pesticides. Only farmworker 15 showed less exposure to pesticides with the variation of both AChE and BChE less than 5% during two recruitments. The field-testing results demonstrated the feasibility of the nanosensor-smartphone platform for onsite rapid and accurate measurement of subclinical exposures where AChE and/or BChE inhibitions are less than 20%.

## Conclusions

In summary, we developed a highly stable, reagentless, low-cost, quantitative, and portable smartphone/resistive nanosensor platform for onsite biomonitoring of exposure to OP pesticides from a finger-stick whole blood sample. The proton-sensitive CS/MWCNT/PAnNF nanocomposite film demonstrated exceptional sensitivity, reproducibility, and stability for POC temporal AChE/BChE activity measurements through ACh/BCh hydrolysis, respectively. Integration of anti-interference pretreatments and substrates within a GF pad system provided simple and processing-free measurements. Furthermore, the integrated mobile application allows for efficient resistive data processing, storage, tracking, and sharing of ChE results. Finally, substantial validation of the nanosensing platform with standard methods highlights its potential for the efficient and simple biomonitoring of acute and chronic low-level OP pesticide exposure.

## Supporting Information Available

Additional experimental details, materials, and methods, including reagents and materials, instrumentation, MWCNT/PAnNF synthesis, preparation of human cholinesterase-spiked and pesticide-spiked whole blood samples, validation of the nanosensor with radiometric and Ellman’s method, additional results including Ellman’s calibration curve, nanosensor-smartphone testing protocol, MWCNT/PAnNF characterization, MWCNT/PAnNF conductivity under different proton concentrations, effect of dedoping extent on nanosensor response, nanosensor stability using NaCl, AChE measurements in different whole blood components, RSD values for nanosensor versus Ellman’s, and comparison of nanosensing platform with existing biosensors (PDF)
