# Supplementary material for: An Integrated Nanosensor/Smartphone Platform for Point-of-Care Biomonitoring of Human Exposure to Pesticides
Source: Anal Chem. 2025 Apr 25;97(18):9701–12. doi: 10.1021/acs.analchem.4c06421 (PMC12079637; doi:10.1021/acs.analchem.4c06421)
Supplement: Supplementary file 1 — ac4c06421_si_001.pdf [file ac4c06421_si_001.pdf]

Supporting Information:

An integrated nanosensor/smartphone platform for point-of-care biomonitoring of human exposure to pesticides

Hussian Maanaki[1,2], Letice Bussiere[2], Aleksandr Smirnov[1], Xiuxia Du[1,7], Yu Sun[2], Thomas A. Arcury[3], Phillip Summers[3], Landon Butler[4], Carey Pope[4], Anna Jensen[5], Gregory D. Kearney[6], Joshua T. Butcher[4], and Jun Wang[1,2,7*]

[1]*Department of Bioinformatics and Genomics, University of North Carolina at Charlotte, Charlotte, NC, 28223, USA*

[2]*Nanodiagnostic Technology, LLC, Kannapolis, NC, 28081, USA*

[3]*Wake Forest University School of Medicine, Winston-Salem, NC, 27157, USA*

[4]*Department of Physiological Sciences, Oklahoma State University, Stillwater, OK, 74078, USA*

[5]*North Carolina Farmworkers Project, Benson, NC, 27504, USA*

[6] *Department of Public Health, East Carolina University, Greenville, NC 27834, USA*

[7]*Center for Environmental monitoring and Informatics Technologies for Public Health, University of North Carolina at Charlotte, Charlotte, NC, 28223, USA*

* To whom correspondence should be addressed.

E-mail: jun.wang@charlotte.edu; Phone: +1-704-687-7391

Table of Contents

Materials and Methods **3**

1. **Reagents and Materials** 3
2. **Instrumentation** 3
3. **Synthesis of MWCNT/PAnNFs** 3
4. **Preparation of heat-treated whole blood spiked with human cholinesterase** 4
5. **Preparation of in-vitro pesticide-spiked whole blood samples** 4
6. **Validation of nanosensor with radiometric method.** 4
7. **Validation of nanosensor with Ellman's method** 4

Supplementary Figures **5**

Fig. S1. Calibration curve for human AChE using Ellman's Method. 5

Fig. S2. Testing protocol of the nanosensor-smartphone platform 5

Fig. S3. Characterization of MWCNT/PAnNF nanocomposites and calibration curve for determining dispersion concentration. 6

Fig. S4. Response of nanosensor with various pH solutions. 6

Fig. S5. Response of biosensor with varying extents of dedoping. 7

Fig. S6. Resistance stability of nanosensor upon addition of whole blood vs. NaCl 7

Fig. S7. Measurement of AChE in various blood media 8

Fig. S8. Boxplot showing the RSD values for in-vitro spiked whole blood sample using Nanosensor and Ellman's Method. 8

Supplementary Tables **9**

Table S1. Comparison of MWCNT/PAnNF resistive biosensor and other biosensors/assays for measurement of human cholinesterase 9

References **10**

Materials and Methods

1. Reagents and Materials

Paraoxon-Methyl (PM), Diazinon-Oxon (DZO), phosphate buffer saline (PBS), Tween-20, chitosan (CS), acetylcholinesterase (AChE) from Electrophorus electricus, human erythrocyte (H-AChE), human plasma butyrylcholinesterase (H-BChE), acetylcholine (ACh), butyrylcholine (BCh), carboxylic acid functionalized multiwalled carbon nanotubes (MWCNT), aniline, ammonium persulfate (APS), hydrochloric acid (HCl), sulfuric acid (H_2SO_4), hydrogen peroxide (H_2O_2), acetone, magnesium chloride ($MgCl_2$), calcium chloride ($CaCl_2$), BW284c51 AChE inhibitor, and pH test strips were purchased from Sigma-Aldrich (St. Louis, MO). All chemicals used in this study were analytical reagent grade. All stock solutions were prepared using deionized (DI) water purified with the Nanopure System (Barnstead, Kirkland, WA). Enzyme stocks were prepared in 1 mM PBS pH 7.4 and diluted in purified deionized water prior to use.

Disposable screen-printed gold interdigitated electrodes (AuIDEs), consisting of 90 pairs of interdigitated fingers with 10-micron widths and gaps were purchased from MicruX Technologies (Gijón, Asturias, Spain). Adapters for connecting AuIDEs to a multimeter were also purchased from Micrux Technologies. Glass fiber (GF) pads for reagent loading were purchased from Sigma-Millipore, Inc. (Temecula, CA). Microcapillary blood collection tubes with lithium heparin (green) were purchased from RAM scientific (Nashville, TN).

2. Instrumentation

Bluetooth Owon digital meter and an android smartphone with APP were used to measure the resistance of a nanosensor over time (two reads/second). The APP can process and analyze the raw resistance data and display the result for each test. JEOL JEM 2100 LaB6 Transmission Electron Microscope (TEM) was used to characterize MWCNT/PAnNF nanocomposite. A diluted suspension was loaded on a copper grid and allowed to dry at room temperature prior to imaging. Nanodrop 2000 was used to measure absorbance of the MWCNT/PAnNF suspensions prior to drop-casting to ensure reproducible films. Digital Pro Plus TH-SPQXJ-20A ultrasonic water bath was used to heat denature human ChE in whole blood samples prior to spiking with known concentrations of human AChE or BChE and for dispersion MWCNT/PAnNF suspensions. Perkin Elmer Liquid Scintillation Analyzer Tri-Carb 2810 TR was utilized to measure the concentration of AChE and BChE in whole blood samples using the radiometric method. BioTek Synergy LX microplate reader was used for running Ellman’s method. A VWR incubator was used to determine the effect of temperature on nanosensor signal generation. Nanosensors and reagent-loaded GF pads were placed in a vacuum desiccator with silicon beads from SP Scienceware.

3. Synthesis of MWCNT/PAnNFs

MWCNT/PAnNF was synthesized utilizing a simple and facile chemical approach according to literature[1] and is the same as we previously published[2]. To reiterate, 20 mL 1.0 mg/mL MWCNT suspension was prepared by adding 20 mg MWCNT to 20 mL HCl and the suspension was sonicated to disperse MWCNT in the solution. Then, the different amount of MWCNT suspension (e.g., 0, 1.5 mL, 3 mL, 7.6 mL of 1.0 mg/mL) were sequentially added to 4 duplicates of 10 mL of HCl containing 0.255 g aniline, respectively. The mixtures were shaken and sonicated to make uniform suspensions. Immediately, 10 mL of 1.0 M HCl containing 182.56 g ammonium peroxydisulfate was added to the above suspensions, shaken vigorously for 30 seconds, and left undisturbed overnight at room temperature to avoid aggregate formation due to constant stirring/sonication [3]. It was observed that the color of the solution gradually changed from clear to deep green without MWCNT and from black to dark green with MWCNT. As-prepared PAnNFs and

MWCNT/PAnNF suspensions (~wt.%, 0.5, 1.0, and 2.5) were dialyzed against water using dialysis membrane tubing to remove HCl and excess reactants; thus, resulting in partially dedoped CNT/PAnNFs (color change from dark green to light green). All PAnNFs and MWCNT/PAnNFs suspensions were characterized using TEM, UV-Vis, and a resistance meter and stored in a dark room at room temperature.

4. Preparation of heat-treated whole blood spiked with human cholinesterase

Human erythrocyte AChE (H-AChE) and human plasma BChE (H-BChE) were spiked in heat-treated whole blood to establish the calibration curves. Briefly, 300 µL of whole blood sample (Zen-Bio, Inc) were first heated in a water bath at 54 °C for 1 hour to denature all AChE and BChE, while maintaining the whole blood matrix for testing[4]. Whole blood AChE and BChE denaturation was confirmed using both Ellman's assay and the nanosensor, indicating complete loss of ChE activity. Subsequently, a series of standard H-AChE or H-BChE were spiked into the heat denatured human whole blood at concentrations within the range of 0-18 U/mL H-AChE and 0-5 U/mL H-BChE, respectively.

5. Preparation of in-vitro pesticide-spiked whole blood samples

Four whole blood samples from four healthy people (Zenbio, Inc) were spiked with the organophosphate (OP) pesticides, Paraoxon-Methyl (PM) (0-820 ppb) or Diazinon-Oxon (DZO) (0-4.2 ppm) to achieve approximately 0, 10%, 20%, 30%, 50% and 80% AChE inhibition in whole blood. Blood samples without spiked-in pesticides served as controls. In total, there are 4 controls, 20 PM-spiked samples, and 20 DZO-spiked whole blood samples. All samples were split in halves; one half was stored at -20 °C before use and the other half were shipped to Oklahoma State University with dry ice for validation using the standard radiometric method[5, 6]. Whole blood AChE and BChE measurements were performed in triplicates using the nanosensor (total ~300 tests).

6. Validation of nanosensor with radiometric method.

Validation of nanosensor measurements for in-vitro spiked whole blood samples and field testing was performed using a standard radiometric method. The radiometric method was conducted with a modification[6] of the Johnson and Russels method for cholinesterase measurements[5]. Briefly, samples were first diluted 30-fold in 50 mM pH 7.0 PBS. Next, 60 µL of PBS or 25 mM acetylcholinesterase inhibitor BW284c51 in PBS is added to the scintillation vials for AChE or BChE measurements, respectively. To the vials, 20 µL of 30-fold whole blood sample is added, vortexed, and immediately placed into a water bath at 26 °C for 60 seconds prior to adding 20 µL 5 mM acetylcholine iodide substrate in PBS. The reactions were then incubated for 7.5 minutes and subsequently terminated by the addition of 100 µL acidic stop solution (1 M chloroacetic acid/0.5 M NaOH/2 M NaCl). Upon termination, 5 mL of organic counting scintillation fluid is added to each vial, vortexed, and left undisturbed overnight for counting the following day. Total cholinesterase activity was determined as the activity in samples without AChE-specific inhibitor, while butyrylcholinesterase (BChE) activity was determined as the activity with the inhibitor. AChE activity was calculated as the difference between total and BChE activity.

7. Validation of nanosensor with Ellman's method

Ellman's method was utilized to further evaluate the efficacy of the nanosensor for OP pesticide exposure assessment through blood cholinesterase measurements. Specifically, Ellman's method was used to measure the acetylcholinesterase activity of 28 of 48 in-vitro OP-pesticide spiked samples. First, a standard protocol was used as previously published[7], and the calibration curve was developed as shown in Figure S8. Briefly, to prepare the standard calibration curve, human acetylcholinesterase was diluted to concentrations of 18, 9, 4.5, 2.5, and 1.125 U/mL in 10 mM PBS pH 7.2. Following that, it was previously determined that blood samples need to be diluted 40-fold to reduce background interference. As such, the standard aforementioned concentrations were also diluted 40-fold to better mimic the final assay protocol under real sample testing.

To run Ellman's method, the procedure is as follows: 240 µL 50 mM PBS pH 7.2, 20 µL 0.4 mg/mL DTNB, and 20 µL 40-fold diluted whole blood was added to each well of a microplate. Following that, 20 µL 2.0 mM AtCh was added to each well of the microplate and shaken for 10 minutes. During this incubation period, AtCh is quickly hydrolyzed by AChE and produces thiols, which subsequently react with DTNB (Ellman's reagent) to form a chromogen that can be measured at 412 nm using a microplate reader. In parallel, background signals were obtained by replacing the AtCh with 50 mM PBS pH 7.2 for each whole blood sample and also shaken for 10 minutes. As such, the final corrected colorimetric signal was determined using the following equation:

$$Absorbance_{corrected} = Absorbance_{with\ AtCh} - Absorbance_{without\ AtCh}$$

Finally, AChE activity was determined by reflecting the corrected absorbance against the standard calibration curve provided in Figure S8.

Supplementary Figures

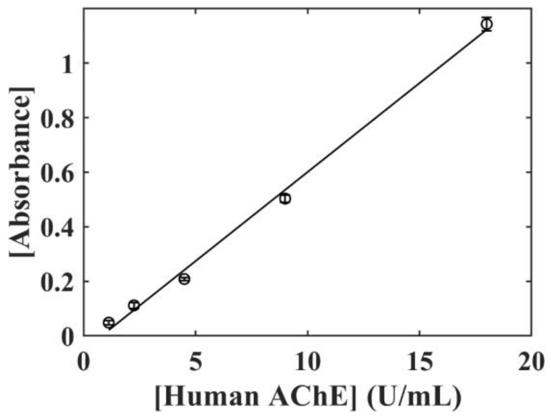

Fig. S1. Calibration curve for human AChE using Ellman's Method. (y = 0.065305x – 0.0525) under the above-mentioned protocol.

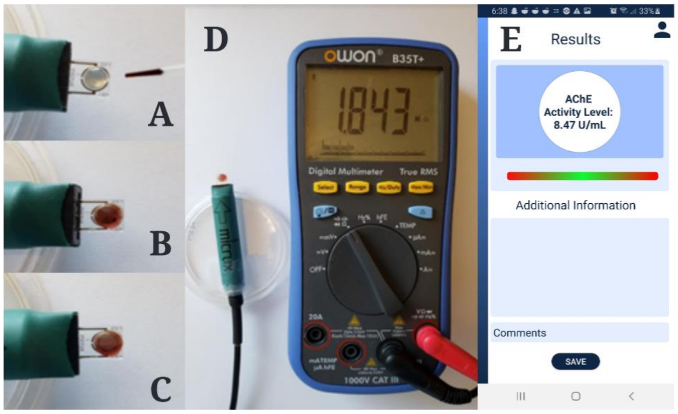

Fig. S2. Testing protocol of the nanosensor-smartphone platform. **[A]** Blood sample application. [B] Outer-pad pre-treatment incubation. **[C]** Inner pad response generation. **[D]** Nanosensor connected to the OWON B35T handheld Bluetooth multimeter. **[E]** The mobile app displays results after completing the test.

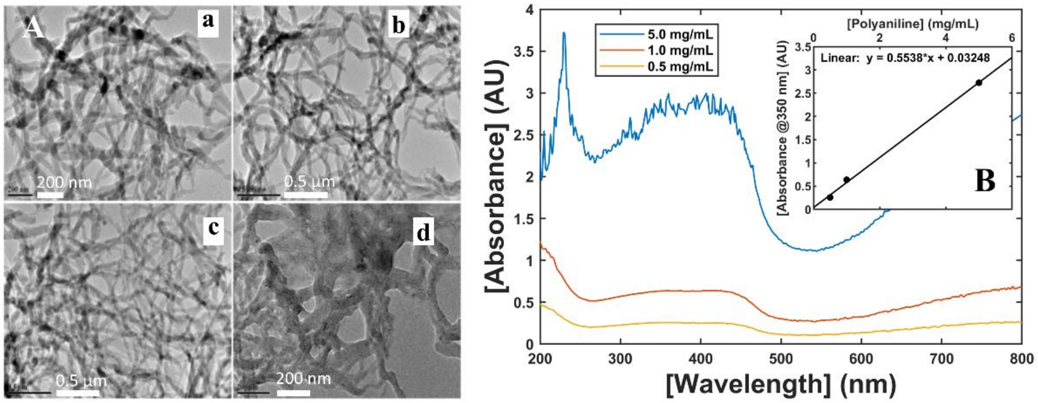

Fig. S3. Characterization of MWCNT/PAnNF nanocomposites. **[A]** Transmission electron microscopy images of MWCNT/PAnNF nanocomposites with **(a)** 0% MWCNT, **(b)** 0.5% MWCNT, **(c)** 1% MWCNT, and **(d)** 2.5% MWCNT. **[B]** UV-Vis spectra for PAnNFs at 0.5, 1.0, and 5.0 mg/mL . Inset calibration curve is provided using the measured absorbance at ~350 nm.

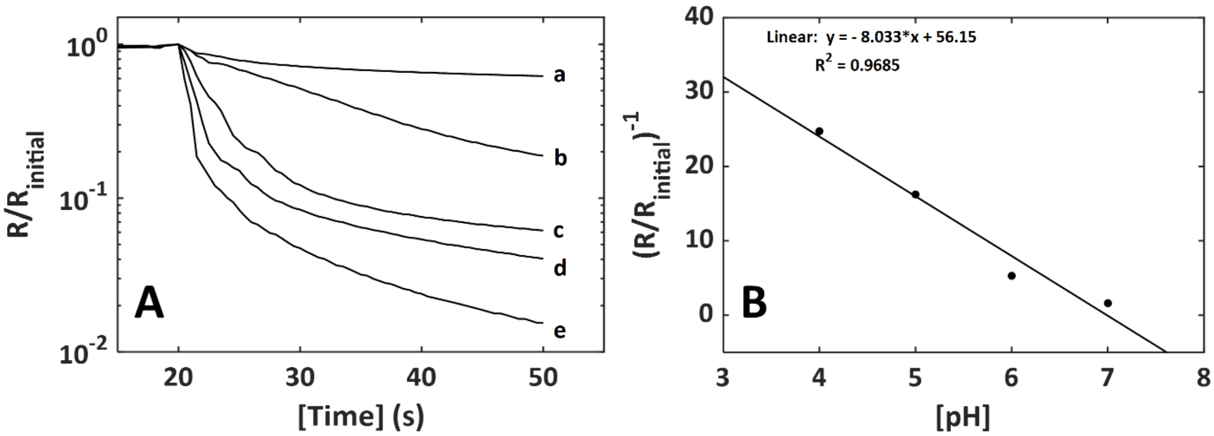

Fig. S4. Response of nanosensor with various pH solutions. **[A]** Normalized response curves of nanosensor with HCl at varying pH: **(a)** 7.0, **(b)** 6.0, **(c)** 5.0, **(d)** 4.0, and **(e)** 3.0. **[B]** Linear fit of nanosensor responses at 50 seconds as a function of pH between 4 and 7.

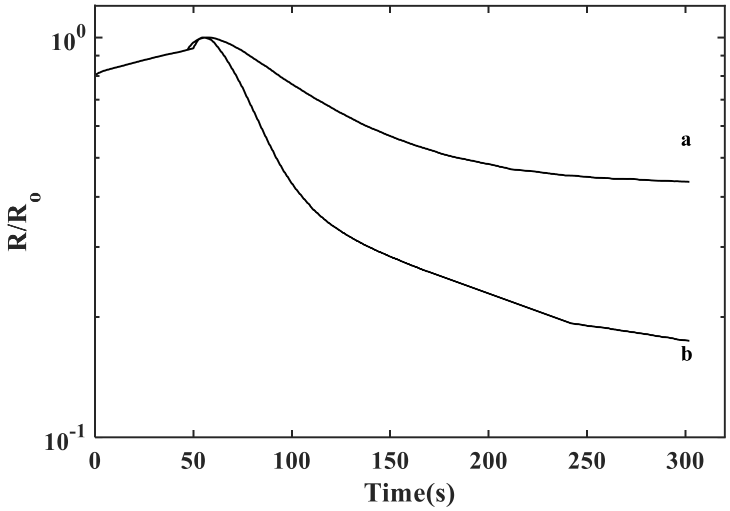

Fig. S5. Response of biosensor with varying extents of dedoping: **(a)** 4 MΩ, and **(b)** 20 MΩ. Biosensor parameters used were: 4 µL MWCNT/PAnNF (Abs. 0.65 @ 350nm), 4 µL 1:1 0.05% chitosan and 0.1323 U eel AChE, and 10 µL 3 mM ACh pre-dried on GF pad.

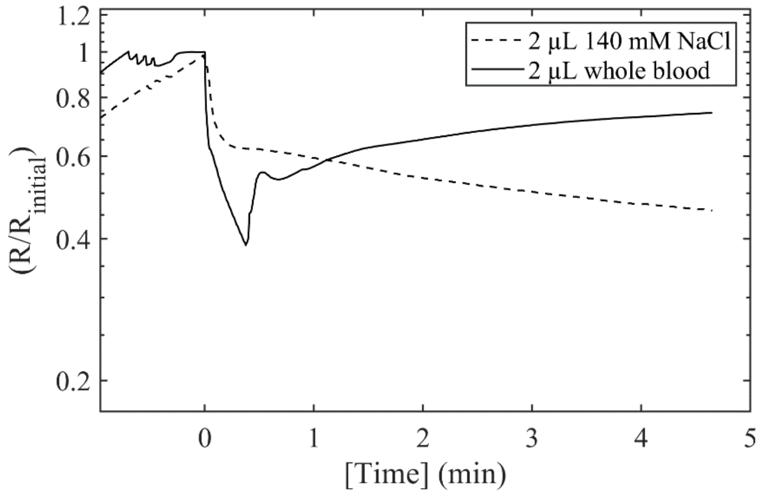

Fig. S6. Resistance stability of nanosensor upon addition of 2 µL whole blood vs. 2 µL equivalent NaCl concentration to 15 µL of DI water on surface.

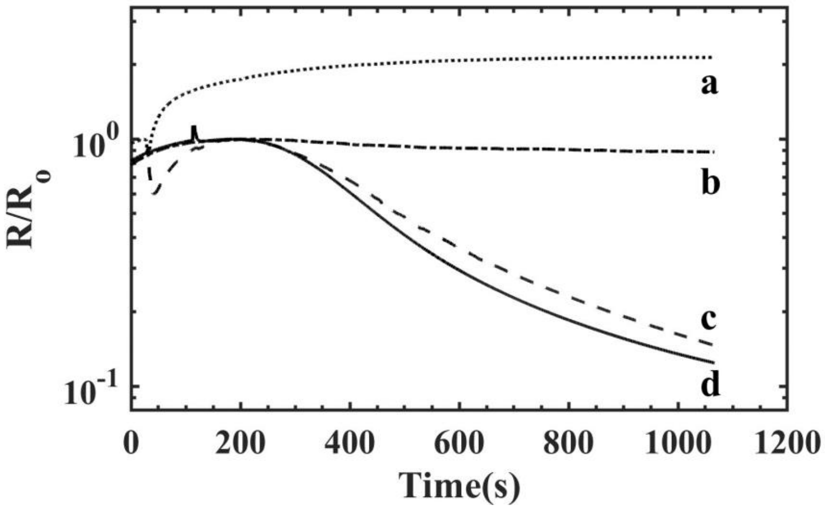

Fig. S7. Measurement of AChE in various blood media: **(a)** control with no ACh, **(b)** plasma, **(c)** RBCs, and **(d)** whole blood. Responses generated with 2 μL whole blood and equivalent amounts of plasma and RBCs. Substrate GF pad loaded with 3 μL 15 mM ACh.

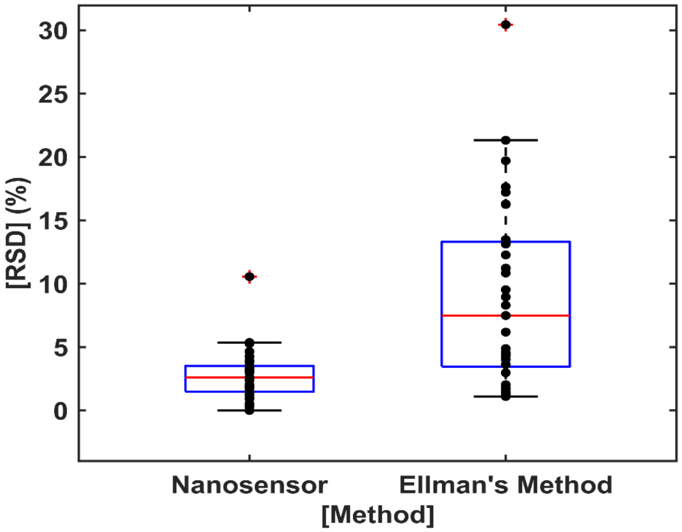

Fig. S8. Boxplot showing the RSD values for all samples (n=48) using the nanosensor and 28 of 48 samples using Ellman's Method. Each sample with each method are tested in triplicates.

Supplementary Tables

Title	Sample type	Target analyte(s)	Dynamic range	Limit-of-detection	Reproducibility	Real Sample Testing	Reference
**An integrated nanosensor/smartphone platform for point-of-care biomonitoring of human exposure to pesticides**	**Whole Blood**	**AChE and BChE**	**(0.2 - 18.0 U/mL, AChE), (0.1 - 5.0 U/mL, BChE)**	**(0.251 U/mL, AChE), (0.114 U/mL, BChE)**	**Same as real sample testing**	**in-vitro AChE and BChE (average RSD = 2.75%, n = 300) Field AChE and BChE Testing (average RSD = 2.55%, n = 270)**	**This work**
Novel "turn on-off" paper sensor based on nonionic conjugated polythiophene-coated CdTe QDs for efficient visual detection of cholinesterase activity	Serum	AChE	0.0167 - 1.17 U/mL	(.00213 U/mL)	RSD_real sample = 3.32 to 4.88 (n = unknown)	Low concentration AChE-spiked serum samples. (RSD = 3.32 to 4.88%), (recovery rate = 107 to 112%), n = unknown	{Ou et al., 2020}
Precision medicine in Alzheimer's disease: An origami paper-based electrochemical device for cholinesterase inhibitors	Whole blood - platform separates it to plasma	BChE	(2 - 12 U/mL, BChE)	N/A	RSD = 4.2%	Many whole blood samples tested with various Alzheimer's drugs. No comparison with standard methods.	{Caratelli et al., 2020}
A novel cholinesterase assay for the evaluation of neurotoxin poisoning based on the electron-transfer promotion effect of thiocholine on an Au electrode	Whole blood, RBCs, and plasma	AChE and BChE	(Approx 0.01 - 0.1 U/mL, AChE)	N/A	(0.738 to 18.5 % for 10 samples, n=3)	Okay correlations with standard method using the three sample types. Measurement principle is simple.	{Shimada et al., 2019}
Simultaneous detection of dual biomarkers from humans exposed to organophosphorus pesticides by combination of immunochromatographic test strip and ellman assay	Plasma	BChE, active and phosphorylated	(0.22 - 3.58 nM, total BChE), (0.22 - 7.17 nM, active BChE)	(0.10 nM, total and phosphorylated BChE)	RSD = ± 5%	Validated with 124 human plasma samples. Pearson correlation between strip and LC/MS/MS was 0.85, $p<0.001$	{Yang et al., 2018}
A reagent-free paper-based sensor embedded in a 3D printing device for cholinesterase activity measurement in serum	Serum	BChE	(0 - 12 U/mL, BChE)	(0.5 U/mL, BChE)	RSD < 5%	Two serum samples tested. Obtained recovery rates of 75 and 83%. No comparison with standard methods was performed.	{Scordo et al., 2018}
Assay of serum cholinesterase activity by an amperometric biosensor based on a co-crosslinked choline oxidase/overoxidized polypyrrole bilayer	Serum	AChE and BChE	(0 - 0.2 U/mL, AChE), (0 - 0.6 U/mL, BChE)	(0.005 U/mL, AChE and BChE)	N/A	4 serum samples tested and compared with standard Ellman's method. Results show no significant differences between the two methods (95% CI)	{Ciriello et al., 2018}
Voltammetric assay of butyrylcholinesterase in plasma samples and its comparison to the standard spectrophotometric test	Plasma	BChE	N/A	(1.14 pkat, BChE)	N/A	Spiked mouse plasma samples with 7 concentrations of carbofuran (n=5). High correlation (R-squared = 0.996) between absorbance of standard spectrophotometric method and square wave peak of electrochemical method.	{Pohanka et al., 2014}
Electrochemical Detection of Dual Exposure Biomarkers of Organophosphorus Agents Based on Reactivation of Inhibited Cholinesterase	RBC membrane only	AChE	(0.5 - 5.0 nM, AChE),	(0.5 nM, AChE)	< 8% RSD	Using RBC membrane samples, an RSD of 8% was observed. Furthermore, consistent results were obtained in comparison to Ellman's method; however, only three samples were tested.	{Ge et al., 2013}
Integrated Lateral Flow Test Strip with Electrochemical Sensor for Quantification of Phosphorylated Cholinesterase: Biomarker of Exposure to Organophosphorus Agents	RBC membrane only	AChE	(0.05 - 10.0 nM, AChE)	(0.02 nM, AChE)	< 8% RSD	Biosensor was evaluated using RBC membrane samples. Average RSD for 3 different samples (n=3) was less than 8%.	{Du et al., 2012}
A Novel Nanoparticle-Based Disposable Electrochemical Immunosensor for Diagnosis of Exposure to Toxic Organophosphorus Agents	Plasma	OP-BChE	(0.1 - 30 nM, OP-BChE)	(0.03 nM, OP-BChE)	4.5% RSD	OP-BChE spiked in human plasma sample. Recoveries ranged from 91.5% to 103.0%. Correlated DFP-spiked plasma samples with DFP-BChE concentrations.	{Lu et al., 2011}
Magnetic Electrochemical Sensing Platform for Biomonitoring of Exposure to Organophosphorus Pesticides and Nerve Agents Based on Simultaneous Measurement of Total Enzyme Amount and Enzyme Activity	N/A	Active and Total BChE	(0.1 - 20 nM, total and active BChE)	(0.05 nM, total and active BChE)	< 8% RSD	N/A	{Du et al., 2011}
Carbon nanotube-based electrochemical sensor for assay of salivary cholinesterase enzyme activity: an exposure biomarker of organophosphate pesticides and nerve agents	Saliva	AChE	(5 pM - 0.5 nM, AChE)	(2 pM, AChE)	< 8% RSD	Measured AChE activity for paraoxon-spiked rat salivary samples. Average RSD for 3 different samples (n=3) was less than 8%.	{Wang et al., 2008}
Highly sensitive electrochemiluminescenc assay of acetylcholinesterase activity based on dual biomarkers using Pd–Au nanowires as immobilization platform	Serum	AChE	(0.025 UL^{-1} - 25 KUL^{-1}, AChE)	(0.0083 UL^{-1}, AChE)	< 5% RSD	Spiked different concentrations of AChE into human blood serum. Recoveries ranged from 95.6% to 101.2 %. RSD (n=3 per sample) ranged from 3.8% to 6.2%.	{Ye et al., 2016}
A Nanozyme- and Ambient Light-Based Smartphone Platform for Simultaneous Detection of Dual Biomarkers from Exposure to Organophosphorus Pesticides	Plasma	Total and Active BChE	(0.05 - 6.4 nM, total BChE), (0.1 - 6.4 nM, active BChE)	(0.025 nM, total BChE), (0.028, active BChE)	< 4% RSD	Ethyl-paraoxon spiked human plasma samples. Recoveries ranged from 94% to 106%. RSD for 3 samples (n=3) was less than 7.01%.	{Zhao et al., 2018}
Nanoparticle-based electrochemical immunosensor for the detection of phosphorylated acetylcholinesterase: an exposure biomarker of organophosphate pesticides and nerve agents	Plasma	OP-AChE	(10 pM - 4 nM, OP-AChE)	(8 pM, OP-AChE)	4.3 % RSD	Human plasma samples spiked with OP-AChE. Recoveries ranged from 106% to 109%. RSD ranged from 3% to 12.8%.	{Liu et al., 2008}
Magnetic electrochemical immunoassays with quantum dot labels for detection of phosphorylated acetylcholinesterase in plasma	Plasma	OP-AChE	(0.3 - 300 ng/mL, OP-AChE)	(0.15 ng/mL, OP-AChE)	11.5 % RSD, performance assessed in plasma	Plasma samples spiked with OP-AChE from 0.01 to 600 ng/mL (11 concentrations, replicates unknown). RSD for the 11 samples was 11.5%.	{Wang et al., 2008}

Table S1. Comparison of MWCNT/PAnNF resistive biosensor and other biosensors/assays for measurement of human cholinesterase[8-23]

References

(1) Huang, J.; Kaner, R. B. A general chemical route to polyaniline nanofibers. *J Am Chem Soc* **2004**, *126* (3), 851-855. DOI: 10.1021/ja0371754.
(2) Maanaki, H.; Xu, T.; Chen, G.; Du, X.; Wang, J. Development of integrated smartphone/resistive biosensor for on-site rapid environmental monitoring of organophosphate pesticides in food and water. *Biosensors and Bioelectronics: X* **2023**, *15*, 100402. DOI: https://doi.org/10.1016/j.biosx.2023.100402.
(3) Li, D.; Kaner, R. B. Shape and aggregation control of nanoparticles: not shaken, not stirred. *J Am Chem Soc* **2006**, *128* (3), 968-975. DOI: 10.1021/ja056609n.
(4) Gorne-Tschelnokow, U.; Naumann, D.; Weise, C.; Hucho, F. Secondary structure and temperature behaviour of acetylcholinesterase. Studies by Fourier-transform infrared spectroscopy. *Eur J Biochem* **1993**, *213* (3), 1235-1242. DOI: 10.1111/j.1432-1033.1993.tb17874.x.
(5) Johnson, C. D.; Russell, R. L. A rapid, simple radiometric assay for cholinesterase, suitable for multiple determinations. *Anal Biochem* **1975**, *64* (1), 229-238. DOI: 10.1016/0003-2697(75)90423-6.
(6) Quandt, S. A.; Pope, C. N.; Chen, H.; Summers, P.; Arcury, T. A. Longitudinal Assessment of Blood Cholinesterase Activities Over 2 Consecutive Years Among Latino Nonfarmworkers and Pesticide-Exposed Farmworkers in North Carolina. *J Occup Environ Med* **2015**, *57* (8), 851-857. DOI: 10.1097/JOM.0000000000000496 From NLM Medline.
(7) Ellman, G. L.; Courtney, K. D.; Andres, V., Jr.; Feather-Stone, R. M. A new and rapid colorimetric determination of acetylcholinesterase activity. *Biochem Pharmacol* **1961**, *7*, 88-95. DOI: 10.1016/0006-2952(61)90145-9.
(8) Ou, Q.; Tawfik, S. M.; Zhang, X. F.; Lee, Y. I. Novel "turn on-off" paper sensor based on nonionic conjugated polythiophene-coated CdTe QDs for efficient visual detection of cholinesterase activity. *Analyst* **2020**, *145* (12), 4305-4313, Article. DOI: 10.1039/d0an00924e.
(9) Caratelli, V.; Ciampaglia, A.; Guiducci, J.; Sancesario, G.; Moscone, D.; Arduini, F. Precision medicine in Alzheimer's disease: An origami paper-based electrochemical device for cholinesterase inhibitors. *Biosens. Bioelectron.* **2020**, *165*, 10, Article. DOI: 10.1016/j.bios.2020.112411.
(10) Shimada, H.; Kiyozumi, Y.; Koga, Y.; Ogata, Y.; Katsuda, Y.; Kitamura, Y.; Iwatsuki, M.; Nishiyama, K.; Baba, H.; Ihara, T. A novel cholinesterase assay for the evaluation of neurotoxin poisoning based on the electron-transfer promotion effect of thiocholine on an Au electrode. *Sensor Actuat B-Chem* **2019**, *298*, 8, Article. DOI: 10.1016/j.snb.2019.126893.
(11) Yang, M. M.; Zhao, Y. T.; Wang, L. M.; Paulsen, M.; Simpson, C. D.; Liu, F. Q.; Du, D.; Lin, Y. H. Simultaneous detection of dual biomarkers from humans exposed to organophosphorus pesticides by combination of immunochromatographic test strip and ellman assay. *Biosens. Bioelectron.* **2018**, *104*, 39-44, Article. DOI: 10.1016/j.bios.2017.12.029.
(12) Scordo, G.; Moscone, D.; Palleschi, G.; Arduini, F. A reagent-free paper-based sensor embedded in a 3D printing device for cholinesterase activity measurement in serum. *Sensor Actuat B-Chem* **2018**, *258*, 1015-1021, Article. DOI: 10.1016/j.snb.2017.11.134.
(13) Ciriello, R.; Lo Magro, S.; Guerrieri, A. Assay of serum cholinesterase activity by an amperometric biosensor based on a co-crosslinked choline oxidase/overoxidized polypyrrole bilayer. *Analyst* **2018**, *143* (4), 920-929, Article. DOI: 10.1039/c7an01757j.
(14) Pohanka, M. Voltammetric assay of butyrylcholinesterase in plasma samples and its comparison to the standard spectrophotometric test. *Talanta* **2014**, *119*, 412-416, Article. DOI: 10.1016/j.talanta.2013.11.045.
(15) Ge, X. X.; Tao, Y.; Zhang, A. D.; Lin, Y. H.; Du, D. Electrochemical Detection of Dual Exposure Biomarkers of Organophosphorus Agents Based on Reactivation of Inhibited Cholinesterase. *Analytical Chemistry* **2013**, *85* (20), 9686-9691, Article. DOI: 10.1021/ac402022p.

(16) Du, D.; Wang, J.; Wang, L. M.; Lu, D. L.; Lin, Y. H. Integrated Lateral Flow Test Strip with Electrochemical Sensor for Quantification of Phosphorylated Cholinesterase: Biomarker of Exposure to Organophosphorus Agents. *Analytical Chemistry* **2012**, *84* (3), 1380-1385, Article. DOI: 10.1021/ac202391w.
(17) Lu, D. L.; Wang, J.; Wang, L. M.; Du, D.; Timchalk, C.; Barry, R.; Lin, Y. H. A Novel Nanoparticle-Based Disposable Electrochemical Immunosensor for Diagnosis of Exposure to Toxic Organophosphorus Agents. *Advanced Functional Materials* **2011**, *21* (22), 4371-4378. DOI: 10.1002/adfm.201100616.
(18) Du, D.; Wang, J.; Wang, L. M.; Lu, D. L.; Smith, J. N.; Timchalk, C.; Lin, Y. H. Magnetic Electrochemical Sensing Platform for Biomonitoring of Exposure to Organophosphorus Pesticides and Nerve Agents Based on Simultaneous Measurement of Total Enzyme Amount and Enzyme Activity. *Analytical Chemistry* **2011**, *83* (10), 3770-3777, Article. DOI: 10.1021/ac200217d.
(19) Wang, J.; Timchalk, C.; Lin, Y. Carbon nanotube-based electrochemical sensor for assay of salivary cholinesterase enzyme activity: an exposure biomarker of organophosphate pesticides and nerve agents. *Environ Sci Technol* **2008**, *42* (7), 2688-2693. DOI: 10.1021/es702335y From NLM.
(20) Ye, C.; Wang, M. Q.; Zhong, X.; Chen, S.; Chai, Y.; Yuan, R. Highly sensitive electrochemiluminescenc assay of acetylcholinesterase activity based on dual biomarkers using Pd-Au nanowires as immobilization platform. *Biosens Bioelectron* **2016**, *79*, 34-40. DOI: 10.1016/j.bios.2015.11.096 From NLM Medline.
(21) Zhao, Y.; Yang, M.; Fu, Q.; Ouyang, H.; Wen, W.; Song, Y.; Zhu, C.; Lin, Y.; Du, D. A Nanozyme- and Ambient Light-Based Smartphone Platform for Simultaneous Detection of Dual Biomarkers from Exposure to Organophosphorus Pesticides. *Analytical Chemistry* **2018**, *90* (12), 7391-7398. DOI: 10.1021/acs.analchem.8b00837.
(22) Liu, G.; Wang, J.; Barry, R.; Petersen, C.; Timchalk, C.; Gassman, P. L.; Lin, Y. Nanoparticle-based electrochemical immunosensor for the detection of phosphorylated acetylcholinesterase: an exposure biomarker of organophosphate pesticides and nerve agents. *Chemistry* **2008**, *14* (32), 9951-9959. DOI: 10.1002/chem.200800412 From NLM Medline.
(23) Wang, H.; Wang, J.; Timchalk, C.; Lin, Y. Magnetic Electrochemical Immunoassays with Quantum Dot Labels for Detection of Phosphorylated Acetylcholinesterase in Plasma. *Analytical Chemistry* **2008**, *80* (22), 8477-8484. DOI: 10.1021/ac801211s.
